# Induction of pancreatic neoplasia in the *KRAS*/*TP53* Oncopig

**DOI:** 10.1242/dmm.049699

**Published:** 2023-01-16

**Authors:** Pinaki Mondal, Neesha S. Patel, Katie Bailey, Shruthishree Aravind, Sara B. Cartwright, Michael A. Hollingsworth, Audrey J. Lazenby, Mark A. Carlson

**Affiliations:** ^1^ Department of Surgery, University of Nebraska Medical Center, Omaha, NE 68198, USA; ^2^ Department of Surgery and VA Research Service, Nebraska-Western Iowa Health Care System, Omaha, NE 68105, USA; ^3^ Eppley Institute for Research in Cancer and Allied Diseases, Fred and Pamela Buffett Cancer Center, University of Nebraska Medical Center, Omaha, NE 68198, USA; ^4^ Department of Pathology, University of Nebraska Medical Center, Omaha, NE 68198, USA; ^5^ Department of Genetics, Cell Biology and Anatomy, University of Nebraska Medical Center, Omaha, NE 68198, USA

**Keywords:** Oncopig, Pancreatic cancer, Pancreas, Porcine pancreatic cancer

## Abstract

The 5-year survival of pancreatic cancer (PC) remains low. Murine models may not adequately mimic human PC and can be too small for medical device development. A large-animal PC model could address these issues. We induced and characterized pancreatic tumors in Oncopigs (transgenic swine containing *KRAS*^G12D^ and *TP53*^R167H^). The oncopigs underwent injection of adenovirus expressing Cre recombinase (AdCre) into one of the main pancreatic ducts. Resultant tumors were characterized by histology, cytokine expression, exome sequencing and transcriptome analysis. Ten of 14 Oncopigs (71%) had gross tumor within 3 weeks. At necropsy, all of these subjects had gastric outlet obstruction secondary to pancreatic tumor and phlegmon. Oncopigs with injections without Cre recombinase and wild-type pigs with AdCre injection did not show notable effect. Exome and transcriptome analysis of the porcine pancreatic tumors revealed similarity to the molecular signatures and pathways of human PC. Although further optimization and validation of this porcine PC model would be beneficial, it is anticipated that this model will be useful for focused research and development of diagnostic and therapeutic technologies for PC.

This article has an associated First Person interview with the joint first authors of the paper.

## Teaser

**Summary:** We induced and characterized pancreatic neoplasms in Oncopigs, observing similarities with human pancreatic cancer, and indicating potential use of the model in the development of diagnostics and therapeutics for pancreatic cancer.

## INTRODUCTION

Pancreatic cancer (PC) is the 14th most common cancer and the fourth highest cause of cancer mortality in the United States (McGuigan et al., 2018; Siegel et al., 2021). It is predicted that PC will be the second-highest cause of cancer mortality by 2030 (De Dosso et al., 2021; Rawla et al., 2019). Patients with Stage I PC (tumor less than 2.0 cm with no involved lymph nodes) (National Comprehensive Cancer Network, 2022) have a 5-year survival of 39%, while patients with Stage IV PC (tumor of any size with distant metastases) have a 5-year survival of 11% (Kamarajah et al., 2017). These survival rates have not changed substantially in decades. Despite the development of multimodal therapy, long-term survival is achieved for only 5% of patients (van Roessel et al., 2018).

Murine models have been used in the preclinical study of PC (Olive and Tuveson, 2006; Sharpless and Depinho, 2006; Siolas and Hannon, 2013; Talmadge et al., 2007), including xenografted immunodeficient models, genetically engineered mouse (GEM) models, humanized mice and *in vivo* edited mice. Although murine modeling has produced tremendous advances in the understanding and treatment of cancer in general, the fact remains that only a few percent of drug candidates identified in preclinical studies as potential anti-cancer treatments will be demonstrated to be safe and efficacious in clinical trials and subsequently obtain U.S. Food and Drug Administration (FDA) approval (Sharpless and Depinho, 2006; Kola and Landis, 2004; Reichert and Wenger, 2008; Singh et al., 2012). Murine models may not accurately predict human biology and response to interventions in all circumstances, secondary to differences in genomic sequence and phenotype. Model inaccuracy may contribute to the above low drug approval rate for potential cancer therapeutics (Talmadge et al., 2007; Begley and Ellis, 2012).

Currently, there are no validated large-animal models for PC, although some proof-of-principle studies in swine have been published (Boas et al., 2020; Hendricks-Wenger et al., 2021; Principe et al., 2018). Swine have been shown to be effective models in other fields, including trauma, transplantation, cardiovascular disease and dermatologic conditions (Swindle et al., 2012; Swindle and Smith, 2016). Swine have greater similarity to humans with respect to size, anatomy, physiology, pathophysiological responses and coding sequence than do mice (Kuzmuk and Schook, 2011; Schook et al., 2015a). It, therefore, is conceivable that swine could have greater accuracy in cancer modeling than mice. Importantly, a porcine PC model would permit research and development of imaging instrumentation, interventional catheters and other devices suitable for the human PC patient. Such research and development would be difficult, if not infeasible, in a 20 g mouse.

In 2015, a transgenic swine known as the Oncopig model (OCM) was described (Schook et al., 2015b), which utilized a Cre-Lox system to control expression (CAG promoter driven) of a somatic cassette containing porcine *KRAS*^G12D^ and *TP53*^R167H^. The expressed Kirsten ras proto-oncogene, GTPase (KRAS) mutant was constitutively activated, while the p53 [encoded by tumor protein P53 (*TP53*)] mutant functioned as a dominant negative. Similar to the KPC murine model [*KRAS*^LSL-G12D^;*TP53*^LoxP^;Pdx1-CreER triple mutant model of tamoxifen-inducible pancreatic ductal adenocarcinoma (PDAC)], the OCM can express *KRAS* and *TP53* transgenes through action of Cre recombinase [Cre is expressed endogenously in the mouse under control of the PDX promoter, while the pig requires exogenous adenovirus expressing Cre recombinase (AdCre)] (Hingorani et al., 2005). However, the transgene cassette was integrated into the porcine genome randomly, which is different from the KPC mouse model, at which point mutations were introduced in the endogenous genes. Previously, we attempted induction of PC in the OCM with introduction of relatively low doses of AdCre into the OCM pancreatic duct, but we did not obtain tumor (Remmers et al., 2018 preprint). Herein, we report modification of the AdCre injection protocol, which successfully generated PC in the OCM. These tumors underwent histological, exomic and expressional analysis, with comparison to human PC.

## RESULTS

### Relevant anatomy of the porcine pancreas

**Fig. 1.**
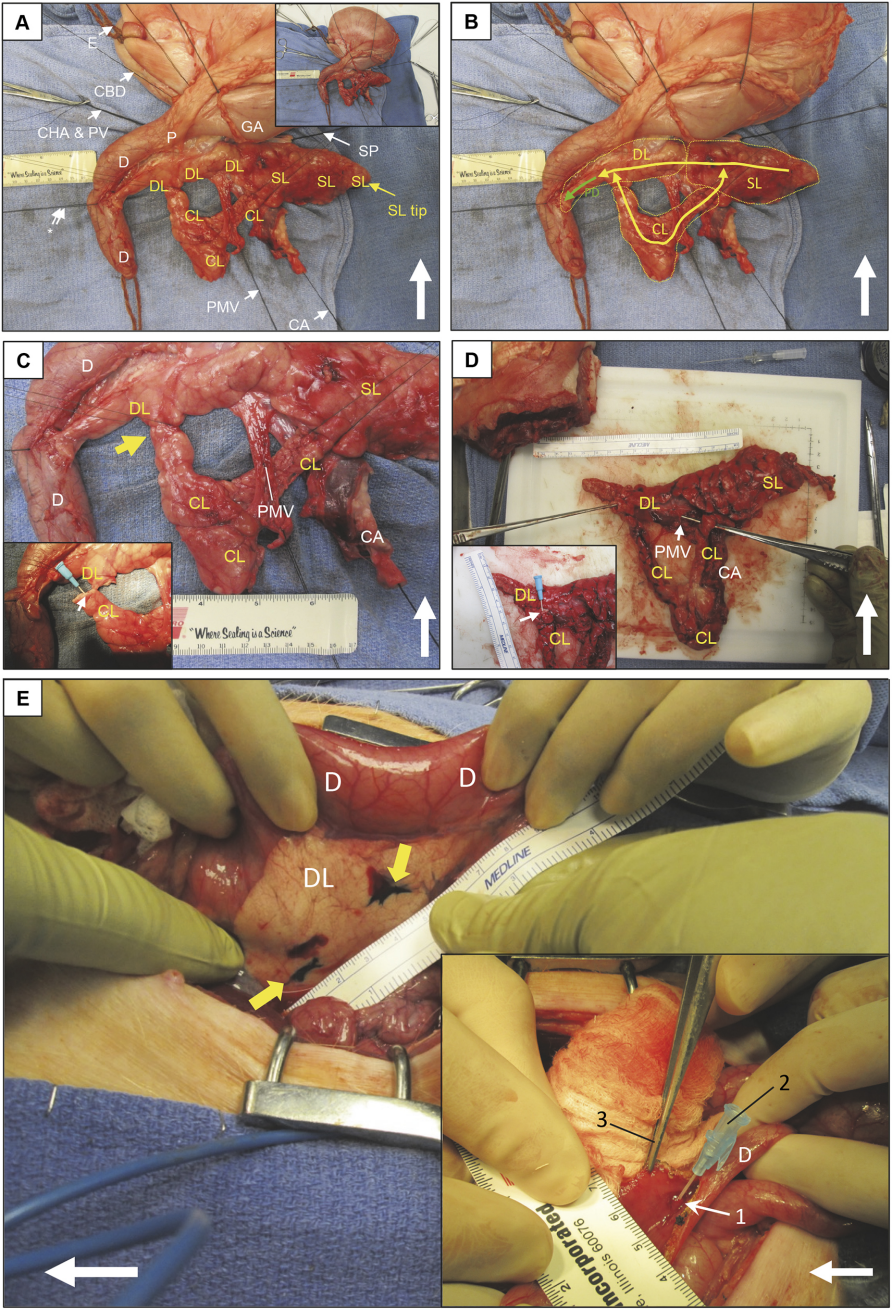
**Porcine pancreatic anatomy and injection techniques.** (A) Anterior aspect; top, cephalad. Double arrow and asterisk indicate the main pancreatic duct entering into duodenum via the pancreatic papilla (not visible). Arrows indicate the structure associated with each label. CA, celiac artery; CBD, common bile duct; CHA & PV, common hepatic artery and portal vein (not visible); CL, connecting lobe; D, duodenum; DL, duodenal lobe; E, esophagus; GA, gastric antrum; P, pylorus; PMV, portomesenteric vein emerging from underneath pancreas; SL, splenic lobe; SP, splenic pedicle. Inset: zoom out view. (B) Same specimen as panel A. Dotted yellow line boundaries indicate the pancreatic lobes. Yellow arrows indicate the approximate course of the pancreatic ductal system. Intersections of the DL, SL and CL form a continuous ductal loop; if interrupted, the system decompresses retrograde into the main pancreatic duct (PD; green arrow). (C) Same specimen, zoomed in. Yellow arrow indicates CL transection for ductal injection. Inset: CL has been transected; 22 g plastic catheter inserted (small white arrow) into CL duct (technique 2 for tumor induction). (D) Similar dissection in another subject, demonstrating circular DL–SL–CL lobar configuration. Inset: cannulization of transected CL duct with a 22 g plastic catheter. (E) Pancreatic parenchymal and main duct injection (technique 2). DL is inked (yellow arrows) for parenchymal adenovirus expressing Cre recombinase (AdCre) injection. Inset: PD accessed through a duodenotomy just opposite to the insertion of the PD; pancreatic papilla (1) then cannulated with 22 g catheter (2) for AdCre injection; DeBakey forceps (3) retracts duodenal wall. Large white arrows in A-E indicate cephalad.

In humans, the pancreas has been labeled with the somewhat arbitrary anatomical regions of ‘head’ (adjacent to the duodenum and to the right of the portomesenteric vein), ‘neck’ (overlying the portomesenteric vein), ‘body’ (to the left of the portomesenteric vein), ‘tail’ (near the splenic hilum) and a variably-present uncinate process that comes off the inferior side of the pancreatic head (Skandalakis et al., 1993). In the pig, however, there are three fairly distinct lobes (Ferrer et al., 2008) that interconnect to form the pancreas: the duodenal, splenic and connecting lobes (Fig. 1A). As described later, this tri-lobar configuration becomes critically important for the porcine PC model. The duodenal lobe of the porcine pancreas (Fig. 1A,B) is an elongated structure situated inferiorly to the pylorus and gastric antrum. The proximal end of the duodenal lobe tapers to a termination on the mesenteric border of the duodenal C-loop, 10-15 cm distal to the pylorus. At this point, the main pancreatic duct traverses the duodenal wall and opens into the duodenal lumen via the pancreatic papilla. The splenic lobe of the porcine pancreas (Fig. 1A,B) is another elongated structure, which initiates at the distal end of the duodenal lobe and terminates near the splenic hilum.

The connecting lobe of the porcine pancreas (Fig. 1A-D) is a structure unique to the pig and has no equivalent in humans. The connecting lobe is a U-shaped structure, which connects the mid-portion of the duodenal lobe to the mid-portion of the splenic lobe. This tri-lobar configuration forms a continuous ring of the porcine pancreas, within which runs a continuous circular ductal system (Fig. 1B). This circular configuration is reminiscent of the arterial circle of Willis, which is located at the base of the human brain. The main pancreatic duct of the pig exits from this circular ductal system within the duodenal lobe (Fig. 1B) and continues proximally to enter the duodenum, as described above. This circular anatomy of the porcine pancreas affords a unique surgical opportunity, in that the ring can be safely interrupted for an intervention (e.g. ductal injection; Fig. 1C-E). After such an interruption, the ductal system will be capable of retrograde decompression away from the interruption point and will not be obstructed.

### Connecting lobe injection in wild-type (WT) pigs

As mentioned in the Introduction, we had previously attempted tumor induction (Remmers et al., 2018 preprint) in the OCM (*n*=5) but did not obtain gross tumor after 4 months of observation (Fig. S2). For those induction procedures, AdCre (the induction reagent) was injected into the main pancreatic duct and parenchyma of the duodenal lobe (technique 1 in the Materials and Methods, descriptive data in Table S2) at a dose of 1×10^8^ plaque-forming units (pfu). Some histological abnormalities were noted (proliferative ductal lesions, Fig. S3; serum test results in Table S3), but none of these appeared to be cancerous. Of note, Schook et al. (Principe et al., 2018) observed a diffuse nodular thickening of the main pancreatic duct, which histologically appeared to be adenocarcinoma in one OCM subject, 12 months after intraductal injection of 4×10^9^[Supplementary-material sup1]pfu of AdCre. We hypothesized that if we increased our dose of AdCre that was injected into the pancreatic duct, then we should obtain ductal cancer in the OCM.

One issue that we noted with our previous group of five OCM subjects (Remmers et al., 2018 preprint) (Fig. S2) was that, after injection of AdCre into main pancreatic duct, the continual flow of pancreatic secretion rapidly flushed the injectate out of the papilla and into the duodenal lumen. This flushing rapidly diluted the AdCre, likely decreasing its efficacy. In order to prevent this flushing, the pancreatic duct would need to be ligated. However, ligation of the main pancreatic duct would obstruct the entire ductal system (Fig. 1A,B), which in swine produces pancreatic exocrine insufficiency (Gregory et al., 2016). We hypothesized that we could instead transect the isthmus region of the proximal connecting lobe (Fig. 1A-D), cannulate and inject the pancreatic duct to the connecting lobe, and then immediately ligate the duct ends with impunity secondary to the circular ductal anatomy of the porcine pancreas (Fig. 1B). Even with the connecting lobe interrupted as above, the entire pancreas could still decompress into the main pancreatic duct (Fig. 1B) after such a maneuver. This ductal injection and ligation would minimize loss of the injectate, which itself would be in contact with the ductal epithelium for a longer duration than had the duct been left open.

In order to determine whether interruption of the isthmus portion of the connecting lobe (along with cannulation of the duct within the connecting lobe) would be feasible and well tolerated, we undertook this procedure in WT domestic pigs (*n*=5; 3-4 months, 30-45 kg; see Table S1 for descriptive data on the porcine subjects), injecting saline distally into the connecting lobe, with ligation of both cut ends of that lobe (i.e. induction technique 2 in the Materials and Methods). This technique effectively interrupted the circular configuration of the porcine pancreas. At operation, the duct within the transected connecting lobe was small (≤1 mm), but could accommodate insertion of a 20-gauge plastic catheter (Fig. 1B,E) and could be flushed easily with 1.0 ml saline. All five pigs tolerated the connecting lobe injection procedure without difficulty. All pigs underwent necropsy 1 month after operation, and there was no evidence of pancreatic inflammation, exocrine insufficiency or other adverse effect from ductal interruption.

### Induction of tumor in OCM subjects: technique 1

We elected to give a higher dose of AdCre (2×10^10^ pfu) in the current experiments than we used previously (1×10^8^ pfu) (Remmers et al., 2018 preprint), i.e. >100-fold increase in the dose of AdCre. We also elected to administer IL-8 as an adjunct with the AdCre; this cytokine has been shown to mobilize the adenoviral receptor to the luminal membrane of epithelial cells, thereby enhancing viral entry (Lutschg et al., 2011; Kotha et al., 2015). The intention of these two changes (high AdCre dose and IL-8) was to increase the chance for successful pancreatic tumor induction in the OCM.

**Fig. 2.**
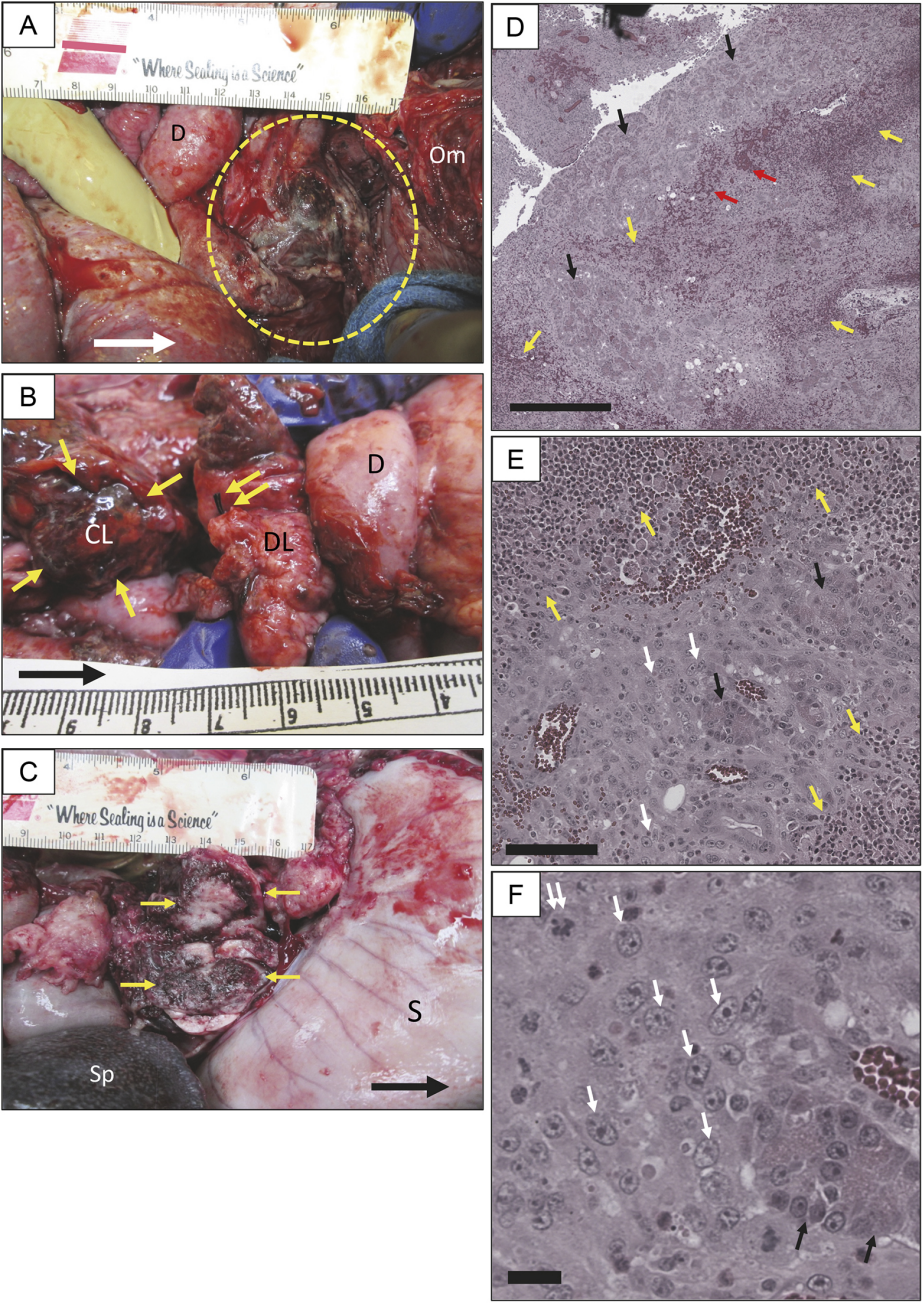
**Induction of Oncopig model (OCM) pancreatic tumor.** (A) OCM necropsy <3 weeks after the tumor induction procedure; dashed yellow line indicates pancreatic phlegmon (contained tumor) at the location of AdCre injection. D, duodenum; Om, omentum. Scale, cm. (B) Another OCM necropsy <3 weeks after tumor induction procedure. Silk suture (double yellow arrow) is at the ligated proximal end of the connecting lobe (CL) at the intersection with the duodenal lobe (DL). Single yellow arrows indicate distal CL remnant (site of tumor). (C) Third OCM necropsy <3 weeks after tumor induction. Transection of CL phlegmon (yellow arrows) demonstrated firm nodular mass (tumor on pathology). Subject had typical gastric outlet obstruction (distended stomach). S, stomach; Sp, spleen. (D) Low-power view (H&E) of CL injection site (pancreatic phlegmon). Cords of inflammatory cells (yellow arrows) intermingled with hemorrhage (red arrows), with residual acini (black arrows). Scale bar: 500 µm. (E) Higher-power view of phlegmon from panel D. Sheets of tumor cells were apparent (white arrows), intermingled with residual acinar structures. Scale bar: 100 µm. (F) High-power view from panel E; individual tumor cells are indicated by white arrows. Double arrow indicates mitotic figure. Scale bar: 20 µm. Large white (A) and black (B,C) arrows indicate cephalad.

The first subject (ID 468; Table 1) underwent induction with technique 1 (i.e. AdCre+IL-8 injection into the main pancreatic duct+injections into the parenchyma of the duodenal lobe); see Fig. 2A. This subject died unexpectedly over a weekend 17 days after the induction procedure. The carcass was refrigerated, and necropsy was performed 72 h later. The subject had gastric perforation with extensive peritoneal soilage (Fig. S4A). The cause of the gastric perforation was gastric outlet obstruction, which in turn was caused by a peripancreatic phlegmon (Fig. S4B). Unfortunately, reasonably intact tissue could not be obtained for histology secondary to the prolonged interval between death and necropsy. Our initial suspicion was that subject 468 developed severe pancreatitis, but not necessarily tumor. The second subject (ID 469; Table 1) underwent a similar tumor induction procedure and underwent euthanasia/necropsy on day 73 (planned endpoint) after an uncomplicated course. There was no gross or microscopic evidence of tumor.

**Table 1.**
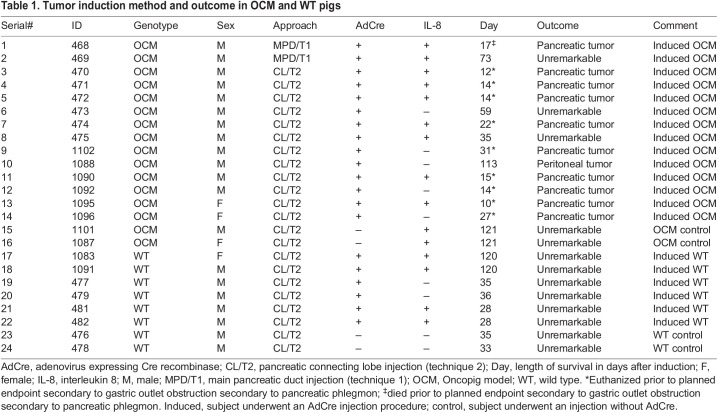
Tumor induction method and outcome in OCM and WT pigs

After these two subjects, we hypothesized that injecting reagents into the main pancreatic duct was not allowing efficient AdCre action secondary to the flushing phenomenon described above. We then attempted connecting lobe injection (technique 2 in the Materials and Methods) for subsequent tumor inductions. We also decided to forgo the concomitant parenchymal injections, secondary to the concern that parenchymal injection would increase exposure of a wide variety of cell types (pancreatic ductal cells, islet cells, fibroblasts, endothelial cells) to AdCre and cause possible transformation. Pleomorphic or sarcomatous tumor induction with nondirected AdCre injection in the OCM has been observed by Schook's group (Principe et al., 2018; Schook et al., 2015b). We reasoned that injection of AdCre directly into a pancreatic duct would keep viral exposure mostly confined to the ductal epithelium and, therefore, produce tumor that would be (mostly) an adenocarcinoma.

### Induction of tumor in OCM subjects: technique 2

Pancreatic tumor induction with AdCre injection into the duct of the connecting lobe (technique 2) was attempted in 12 OCM subjects (Table 1). Only two of these subjects (ID 473 and 475; Table 1) lived to their planned euthanasia date without incident and had no remarkable findings at gross necropsy and no microscopic analysis. Nine of the remaining subjects (75%) had onset of lethargy and decreased feeding at 10-15 days after the induction procedure, with progressive decline that necessitated unplanned euthanasia at a mean of 18±7 days (range 10-31 days). One other subject (ID 1088) underwent unplanned euthanasia on day 113 secondary to respiratory distress, which was not immediately related to study interventions. The gross findings at necropsy in these nine subjects was similar to that in OCM ID 468 above (technique 1; unexpected death): a peripancreatic phlegmon producing gastric outlet obstruction (Fig. 2B; Fig. S4C). In each case, the phlegmonous process had obliterated the region in and around the proximal pancreas; in some cases, there was gross liquefaction. It was difficult to discern anatomical landmarks around the phlegmon. In addition, six of the subjects with tumor had a varying number of extrapancreatic intra-abdominal nodules (<1 cm) studding the surface of various structures (Fig. S4D), including the omentum, mesentery, diaphragm, liver, spleen, parietal peritoneum, stomach and intestines.

The initial concern was that AdCre injection into the connecting lobe of the OCM subjects was inciting severe pancreatitis that produced gastric outlet obstruction, which ultimately was fatal. However, upon cutting into the phlegmon of these subjects, there was a firm, somewhat pale core, which appeared to be tumor (Fig. 2C). Hematoxylin and Eosin (H&E) microscopy of slices taken from the core of the peripancreatic phlegmon from all nine subjects that underwent premature euthanasia demonstrated abundant tumor cells (Fig. 2D-F) with large nuclei and prominent nucleoli. Tumor cell morphology varied from spindle shaped to rounded. Sheets of tumor cells were interspersed with normal-appearing pancreatic acini and the occasional islet, along with areas of hemorrhage and liquefaction necrosis. Areas of tumor were often surrounded by areas of activated lymphocytes (Fig. 2D,E). In addition, there were numerous tumor-infiltrating lymphocytes and macrophages, and occasionally neutrophils and eosinophils. In some subjects, there were numerous multi-nucleated giant cells, which appeared to be engulfing vacuoles of necrotic material.

**Fig. 3.**
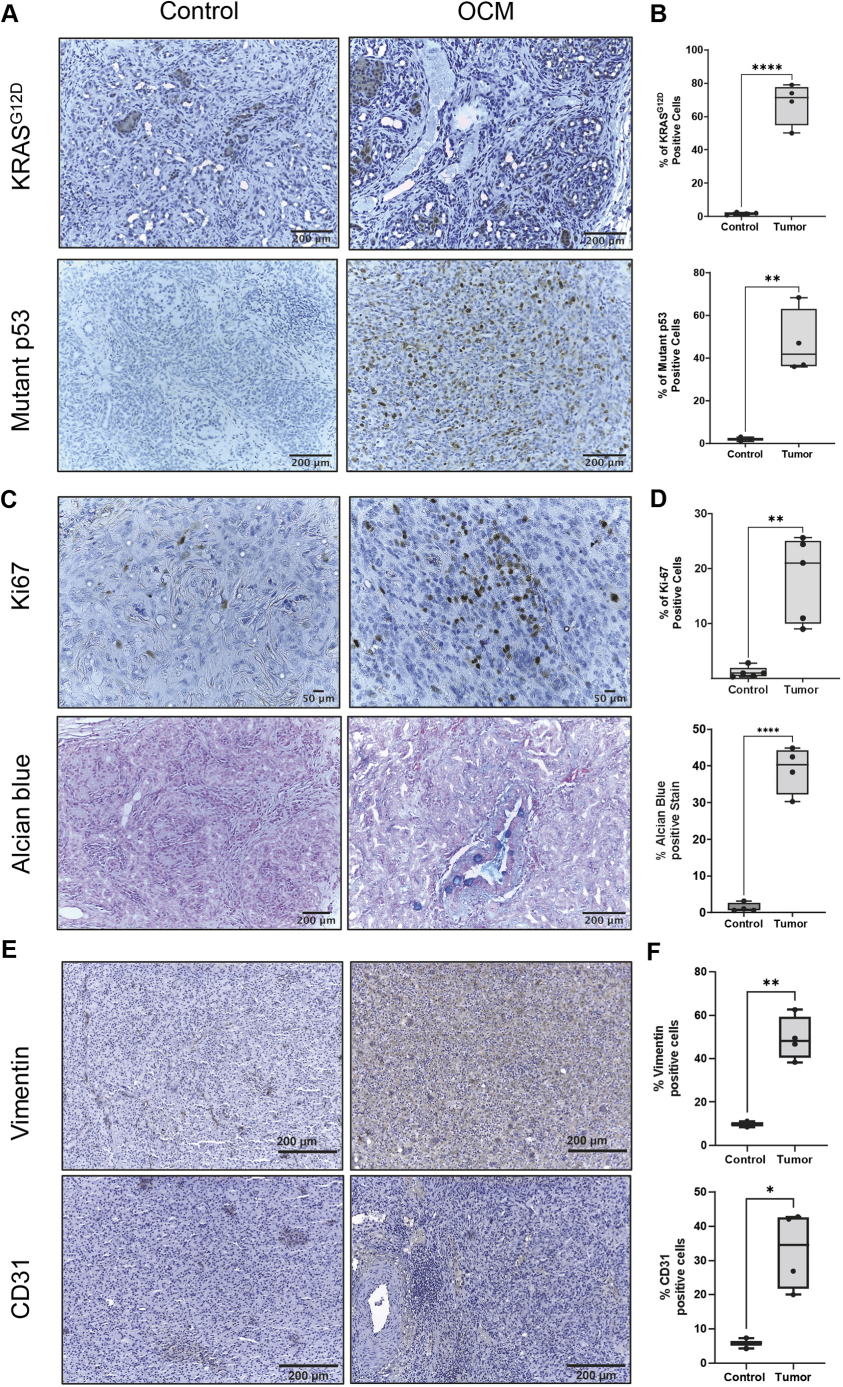
**Immunohistochemistry of tumor-associated proteins and polysaccharide staining in pancreatic tumor from AdCre-induced Oncopigs versus pancreas from wild-type (WT) littermates.** (A-F) Expression and quantification of KRAS (*n*=3, control and OCM) and p53 mutants (*n*=3, control and OCM) (A,B), Ki-67 (*n*=3, control and OCM) and Alcian Blue (*n*=3, control and OCM) (C,D), and vimentin (*n*=2, control and *n*=4, OCM) and CD31 (*n*=2, control and *n*=4, OCM) (E,F). Controls [WT pig with AdCre injection into the connecting lobe (same conditions as OCM treatment)] used for KRAS, mutant p53, Ki-67 and Alcian Blue were subjects 1083, 1087 and 1101. Controls for vimentin and CD31 were subjects 1087 and 1101. Unpaired two-tailed Student's *t*-test, *P<0.05, **P<0.01, ****P<0.0001.

Immunohistochemistry of tumor samples from the peripancreatic phlegmon stained positive for mutant KRAS^G12D^ and mutant p53 (Fig. 3A). Quantification of mutant KRAS^G12D^ and mutant p53 staining demonstrated cellular positivity rates in tumor of ∼70% and ∼40%, respectively; nontreated OCM pancreas had positivity rates close to zero (Fig. 3B). Although the KRAS^G12D^ and mutant p53 express as a bicistronic mRNA in the OCM pigs, the difference in the expression between these two mutant proteins might be due the different ability of the respective antibodies to detect antigens. Tumor sections also had increased Ki-67 (also known as MKI67) and Alcian Blue staining compared to control sections (Fig. 3C,D). These data suggested that OCM pancreatic tumors were more proliferative and had increased acidic mucins and hyaluronic acid deposition into the extracellular matrix, which is consistent with human PC (Kaur et al., 2013; Ogawa et al., 2021; Pergolini et al., 2019). Tumor sections also stained positive for vimentin and CD31 (also known as PECAM1) markers (Fig. 3E,F), which suggested a mixed epithelial phenotype, possibly with epithelial-to-mesenchymal transition (EMT). Quantification of cytokeratin 19 (CK19; also known as KRT19) staining showed no significant difference between tumor and control samples.

In order to compare histopathology between OCM pancreatic tumor and human PDAC, the porcine tumors were immunostained for pan-keratin and CK8, CK18, CK19, CK17 and CK7 (widely used PDAC cytokeratin markers); see Fig. S5. Interestingly, individual tumor cells within OCM tumor did not demonstrate any keratin staining. However, there were some metaplastic-appearing cords of cells within the OCM tumors that did stain (irregularly) for these PDAC markers. This immunopositivity might have represented pre-neoplastic regions in the OCM tumor mass, which may have progressed to neoplasia, becoming undifferentiated in the process. Nevertheless, the OCM pancreatic tumors as analyzed appeared to be poorly differentiated malignant neoplasms (which, incidentally, can be found in human PDAC).

The gross appearance of extrapancreatic nodules that were noted in some OCM subjects (Fig. S4) was consistent with ‘drop’ or contact metastases, being only present on the surface of the involved organ or tissue, and mostly inferior and ventral to the phlegmon. There was no evidence of nodule formation within the body of an organ, such as the liver (as is frequently the case in clinical metastatic PC; National Comprehensive Cancer Network, 2022). Histological evaluation of these extrapancreatic nodules revealed them to be dedifferentiated tumor with a prominent component of activated lymphocytes, both around and within the tumor (Fig. S4). In some cases, these tumor implants showed evidence of invasion into the underlying normal tissue, with disruption of the peritoneum and underlying connective tissue capsule covering the normal tissue.

Subject 1088, which underwent unplanned euthanasia on day 113 secondary to respiratory distress, showed gross evidence of acute pneumonia at necropsy; on histology, the pulmonary alveoli were filled with fluid and acute inflammatory cells. Blood work at necropsy showed an elevated white blood cell count. There also were several nodules (≤1 cm) on the surface of the anterior stomach, but the pancreas was unremarkable. Histological evaluation of the gastric nodules demonstrated dedifferentiated tumor, similar to the above extrapancreatic nodules. There also were sheets of activated lymphocytes present on H&E sections of the liver, spleen and lung. The cause of death was deemed to be pneumonia.

### Control injections in OCM and WT pigs

Injections of tumor-induction agents (AdCre±IL-8) into the connecting lobe (i.e. technique 2) of WT littermates (*n*=6; Table 1) of the OCM subjects were performed to determine whether the procedure or the induction agents would induce any pathology in the background strain of the OCM (Fig. 3). All WT subjects tolerated the injection without difficulty, and there were no remarkable findings at necropsy. H&E evaluation of the connecting lobe of the pancreas (i.e. AdCre injection site) in these subjects revealed minimal chronic inflammation, but otherwise was unremarkable. Immunohistochemical staining of Ki-67 and Alcian Blue was lower in the WT pancreas compared to OCM tumor (Fig. 3C,D).

In order to determine whether some condition unique to the transgenic Oncopig (i.e. not the tumor) was producing severe pancreatitis after the connecting lobe procedure, two OCM subjects underwent IL-8 injection only into the connecting lobe (ID 1101 and 1087; Table 1). These two subjects tolerated the procedure without difficulty and had no remarkable findings at necropsy. Immunohistochemistry of these two OCM controls showed similar staining to WT controls with AdCre injection into the connecting lobe, including positive staining for CK19, vimentin and CD31. The results of the control injections from both WT and OCM subjects suggested that the severe inflammatory response noted in AdCre-treated OCM subjects was secondary to tumor formation and not some other response to the pancreatic injection procedure.

### Effect of IL-8 co-administration

Sorting the data in Table 1 based on AdCre injection with or without IL-8 co-administration revealed pancreatic tumor in 7/9 OCM subjects with IL-8 versus 3/5 subjects without IL-8 (*P*>0.48, chi-square), with early euthanasia at 15±4 and 24±9 days, respectively (*P*>0.2, unpaired two-tailed Student's *t*-test).

### Serum cytokine levels

Some inflammatory cytokines have been shown to be elevated in patients with pancreatitis or PC, and have been implicated in the development and progression of the latter (Roshani et al., 2014). Serum cytokine array analysis performed on three tumor-bearing OCM subjects demonstrated increased expression of IL-1β, IL-6, IL-8 and IL-10 at necropsy compared to pre-induction serum levels (Fig. S6A); the pre-induction versus necropsy levels of other cytokines in the array were not significantly different for subjects with tumor. The changes in the pre-induction versus necropsy levels for the above four cytokines in three WT control subjects were less consistent (Fig. S6B), with upward, downward or no trends being evident. However, statistical comparison of the relative change (pre-induction to necropsy) of each cytokine was not different in the OCM versus WT subjects (Table S4). Pre-induction versus necropsy levels for the other cytokines in the array not shown in Fig. S6B were not significantly different for WT subjects. The baseline (pre-induction) level of each cytokine shown in Fig. S6B was not different in the OCM versus WT subjects (Table S4). Intra- and inter-group variability in the cytokine data were evident for each of these four cytokines, which suggests a variable immune response in the Oncopigs after tumor induction. Use of IL-8 co-administration (to enhance adenoviral entry into epithelial cells) also might have impacted cytokine levels, including IL-8 itself.

### Gene variants in OCM pancreatic tumors associated with human PDAC

**Fig. 4.**
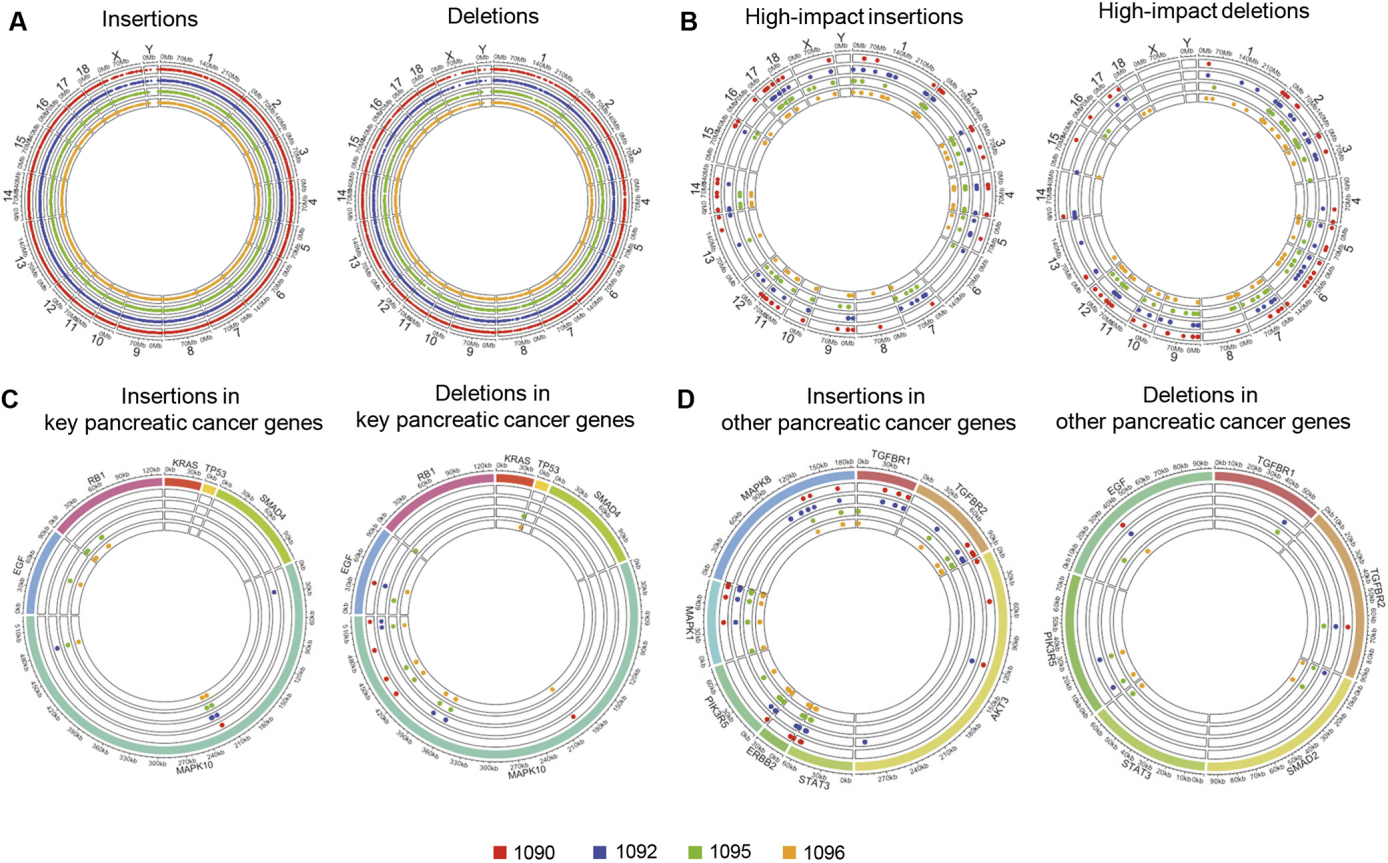
**Exome insertions and deletions in OCM tumors.** All displayed maps compare exome sequence data between a group of four OCM pancreatic tumors (subjects 1090, 1092, 1095 and 1096 from Table 1) and normal porcine pancreas (i.e. Oncopig pancreas without AdCre injection); indels (insertions and deletions) refer to tumor exome with respect to normal. (A) Exome-wide map of all indels. (B) Indels predicted to have an effect on protein expression. (C) Indels involving key genes in human pancreatic cancer (PC). (D) Indels involving other genes related to human PC.

Exome analysis of four Oncopig pancreatic tumors demonstrated insertions and deletions in all chromosomes in all four tumors (Fig. 4A). Functional variations with high prediction to be able to change protein expression were identified through the Ensemble Variant Effect Predictor (Table S5) (McLaren et al., 2016). These high-impact variations on protein expression were present in all the tumors and throughout the exome (Fig. 4B). Variations were found in known PC genes, i.e. *KRAS*, *TP53*, SMAD family member 4 (*SMAD4*) and mitogen-activated protein kinase 10 (*MAPK10*) (Fig. 4C). *KRAS*, the important gene altered in human PC, showed two different types of alterations: deletions and alteration at a known single-nucleotide polymorphism (SNP) position (Table S6). The exome analysis successfully detected the presence of *KRAS ^G12D^* mutation in the Oncopigs (Table S6). Another PC-associated gene in humans, *TP53*, showed two intronic variants in three OCM tumors that are in a known SNP position (Table S6). One mutation in only one pig tumor was found in *SMAD4* (Table S6). The alteration frequency of *KRAS*, *TP53* and *SMAD4* in human PC is presented in Table S7.

Apart from these three common genes of human PC, we identified other genes commonly associated with human PDAC, which were found to be altered by insertions, deletions and functionally relevant variations (Fig. 4D; Table S8). We found deletions in three of four pig tumors in cyclin D1 (*CCND1*), caspase 9 (*CASP9*) and in all samples for Rac/Cdc42 guanine nucleotide exchange factor 6 (*ARHGEF6*) (Table S8). Insertions and deletions were mostly found in *MAPK10*, with occasional presence in epidermal growth factor (*EGF*) and retinoblastoma transcriptional corepressor 1 (*RB1*) (Fig. 4C,D). Together, these preliminary findings suggest that porcine pancreatic tumors showed similar mutational landscape with human PDAC.

### Similarities of gene expression in OCM pancreatic tumor versus human PDAC

**Fig. 5.**
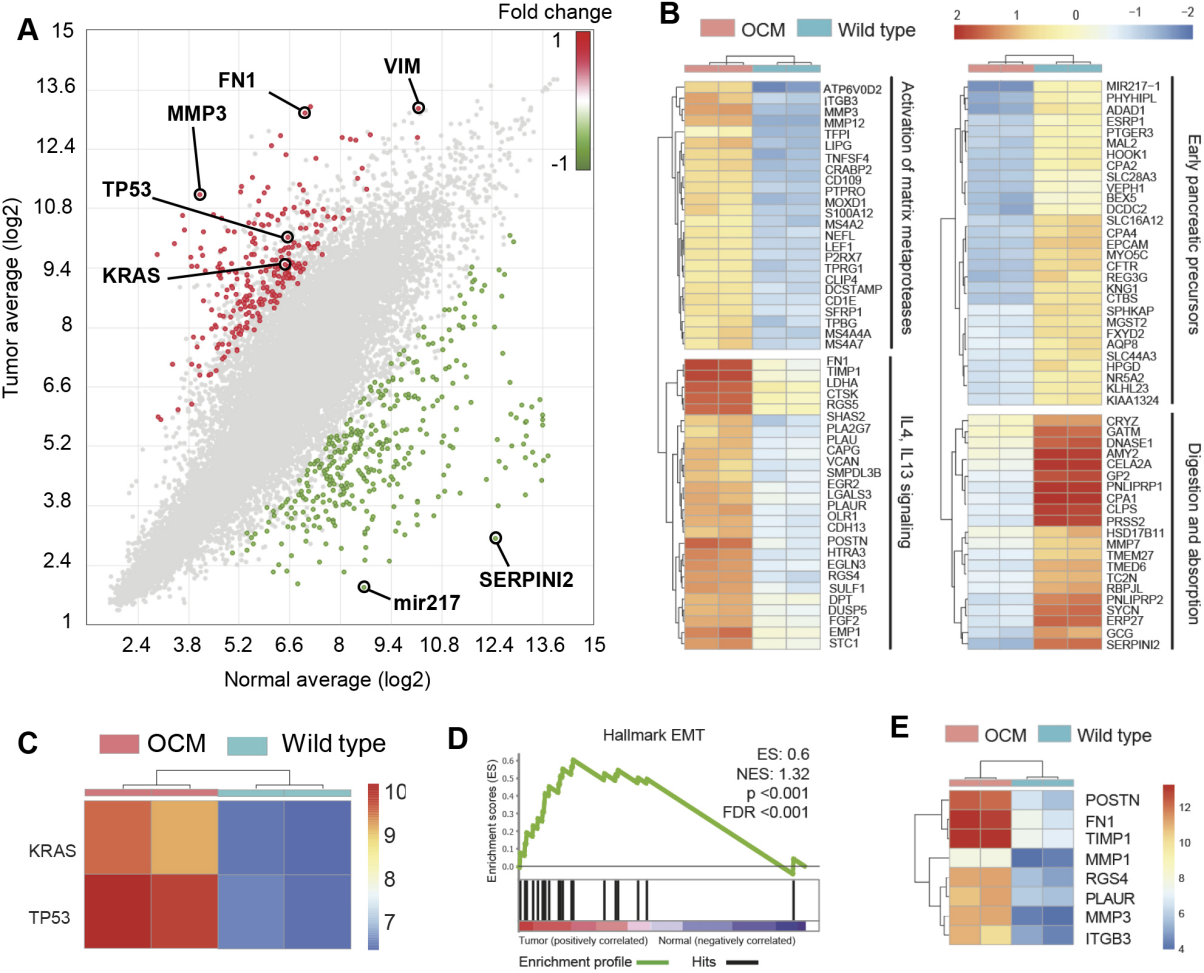
**Transcriptomics of OCM pancreatic tumors versus WT pancreas.** (A) Differential gene expression scatterplot (RNA sequencing) from WT pancreas (*n*=2) and pancreatic tumor (*n*=2). (B) Hierarchical cluster analysis of top 50 upregulated and downregulated genes. (C) *KRAS* and *TP53* expression. (D) Enrichment (ES) score for Hallmark epithelial-to-mesenchymal transition (EMT) genes. FDR, false discovery rate; NES, normalized enrichment score. (E) Differential expression of selected EMT genes.

The transcriptome of OCM pancreatic tumors was compared with that of pancreas of WT littermates. Known human PDAC genes, including *KRAS* and *TP53*, were overexpressed in OCM tumors (Fig. 5A). Genes associated with EMT pathways (Chang et al., 2016), including matrix metalloproteinase 3 (*MMP3*), fibronectin 1 (*FN1*) and vimentin (*VIM*), were among the top overexpressed genes in these tumors (Fig. 5A). Serpin family I member 2 (*SERPINI2*), a tumor suppressor gene downregulated in PDAC and other cancers (Wehr et al., 2011; Bergeron et al., 2010), and microRNA mir-217 (*MIR217*), a potential tumor suppressor gene in PDAC (Zhao et al., 2010), which targets KRAS expression, were among the most underexpressed genes in the OCM tumors (Fig. 5A).

Hierarchical cluster analysis of the top 50 upregulated and top 50 downregulated genes in OCM pancreatic tumors identified four major biological processes that were altered in the tumors (Fig. 5B): (1) matrix metalloproteinases; (2) IL-4/IL-13 signaling; (3) early pancreatic precursors; and (4) digestion and absorption. Matrix metalloproteases and IL-4/IL-13 signaling were upregulated, whereas early pancreatic precursors and digestion/absorption genes were downregulated (Fig. 5B). Various MMPs and IL-4/IL-13 signaling are associated with progression of human PDAC (Slapak et al., 2020; Tjomsland et al., 2016; Shi et al., 2021). In addition, expression of *KRAS* and *TP53* was upregulated in the OCM pancreatic tumors (Fig. 5C). It should be noted that overexpression of *KRAS* and *TP53* may or may not have been secondary to induced transgene expression, as the transcriptome probes detected both WT and mutant gene transcripts. The Hallmark EMT pathway [widely associated with cancer, including PC (Wang et al., 2017; Aiello et al., 2018)] was over-represented in OCM tumors (Fig. 5D). The top enriched (overexpressed) EMT genes in OCM tumors included *MMP1*, *MMP3*, periostin (*POSTN*), *FN1*, tissue inhibitor of metalloproteinase 1 (*TIMP1*), regulator of G-protein signaling (*RGS4*), plasminogen activator urokinase receptor (*PLAUR*) and integrin subunit beta 3 (*ITGB3*); see Fig. 5E.

Analysis of tumor microenvironment gene sets also revealed that TGF-β signaling, matrix metalloproteinases, IL-18 signaling and TH17 cell differentiation signaling pathways were significantly enriched (Fig. S7). Expression of genes [*MMP1*, *MMP3*, *MMP12*, *MMP19*, *TIMP1*, *ITGB3*, *FN1*, interleukin 27 receptor subunit alpha (*IL27RA*) and transforming growth factor beta receptor 1 (*TGFBR1*)] from these pathways, which are known to be upregulated in human PDAC (Slapak et al., 2020; Zhai et al., 2016; Zhu et al., 2019; Ligorio et al., 2019; Chen et al., 2019), was found to be overexpressed in OCM pancreatic tumors (Fig. S7, heatmaps), suggesting similarities between the porcine and human pancreatic tumor microenvironment.

A list of all statistically overexpressed and underexpressed genes in OCM tumors is provided in Table S9.

## DISCUSSION

This proof-of-principle study demonstrates that tumor can be induced in the pancreas of the *KRAS/TP53* Oncopig rapidly and with reasonable reliability. However, the fulminant time course (sometimes <2 weeks), with subjects succumbing to a secondary complication of the tumor (pancreatitis and gastric outlet obstruction), may limit the utility of the model in its present form. For instance, we were unable to detect precursor lesions in the Oncopig tumors, which likely was secondary to the rapid time course and fulminant inflammatory reaction.

The tumor-associated inflammation may have been secondary to an immune response to an acute load of neoantigens in these juvenile, immunocompetent pigs. The immune response against tumor induced within OCM skeletal muscle was previously characterized as an intratumoral infiltration of cytolytic CD8β^+^ T cells (Overgaard et al., 2018), which may be what was observed in the present study. Currently, we do know whether all lesions were solely neoplastic or had some hyperplastic (or metaplastic) components induced by inflammation, as the degree of peritumoral inflammation was high. Although the histology and sequencing demonstrated that tumor was present and recombination had occurred, we cannot exclude the possibility of metaplasia.

Another possible cause of the intense inflammatory reaction could have been a response to adenoviral infection. However, because none of the WT pigs similarly treated with AdCre developed pancreatitis, it is unlikely that adenoviral infection in the Oncopig pancreas produced clinically relevant pancreatitis. One control not performed in this study was to inject the connecting lobe duct of the Oncopig with the adenoviral vector minus Cre, to determine whether there was atypical OCM response to adenoviral infection not present with WT pigs (admittedly, an unlikely possibility).

The clinical course of tumor development and the associated inflammatory reaction might be slowed by decreasing the AdCre dose (with the understanding that lower doses have produced no tumor at all). We are planning a dose-response study to determine whether a minimal dose of AdCre can produce pancreatic tumor with a more practical time course, which would make this model more tractable for researchers. Another option might be to use a different viral vector (e.g. lentivirus), which might result in lower levels of Cre expression. The anti-tumor lymphocytic inflammatory response also might be mitigated by administration of immunosuppression. However, use of immunosuppression would increase the model complexity and might impact model relevance. The fields of tumor immunomodulation and tumor immunoediting currently are under intense study (Wagner and Koyasu, 2019; Schumacher and Schreiber, 2015; Vesely et al., 2011; Zitvogel et al., 2016), and tumor interactions with the immune system in these OCM subjects could be highly relevant (Overgaard et al., 2018).

Co-administration of IL-8 with AdCre was tested in this study to determine whether this chemoattractant could enhance tumor development [IL-8 may increase AdCre entry through the apical membrane of epithelial cells (Lutschg et al., 2011; Kotha et al., 2015)]. Although this study was not designed and did not have adequate numbers to test IL-8 effect, there may have been a trend for IL-8 enhancement of tumor development; to be clear, however, no firm conclusion about IL-8 effect on tumor development can be drawn. Administration of IL-8 alone did not induce pancreatitis.

Subcutaneous tumor growth has been demonstrated in a proof-of-principle study with implantation of human PC cells (Panc01) into the ears of immunodeficient transgenic pigs (*RAG2*/*IL2RG* deficient) (Hendricks-Wenger et al., 2021). Although immunodeficient orthotopic xeno/allograft models have an advantage with respect to xenografting human PC, there are two issues with this approach: (1) the lack of a functional immune system raises issues of clinical relevance (similar to critiques of oncologic studies using immunodeficient mice (Siolas and Hannon, 2013; Zitvogel et al., 2016; Tentler et al., 2012); and (2) husbandry of immunodeficient pigs is complex relative to that of immunocompetent pigs. Hence, it has been our preference to develop an immunocompetent porcine model of PC.

Induction of neoplasia with injection of AdCre into the main pancreatic duct of the Oncopig has previously been described (Principe et al., 2018), but this was a proof-of-principal demonstration in one subject that had microscopic changes only, observed 1[Supplementary-material sup1] year after induction. Another group was able to percutaneously access the Oncopig pancreas under computed tomography guidance and was able to induce tumor that became grossly evident within a month (Boas et al., 2020). Although the percutaneous method avoids a laparotomy, it does not restrict or control the transformation events. This results in nonspecific transformation of many cell types, resulting in pleomorphic tumors.

Tumor pleomorphism has been a described consequence of nondirected injection of AdCre into OCM tissue (Boas et al., 2020; Principe et al., 2018; Mondal et al., 2022). Although it is clear that AdCre injection into the Oncopig at various sites will produce neoplasia, the clinical relevance of resultant pleomorphic tumors is questionable. Our OCM pancreatic tumor induction technique is intended to avoid this nonspecific transformation by directing injection of AdCre into the duct of the surgically isolated connecting lobe, which should produce more specific, epithelial transformation. However, we cannot rule out the possibility that OCM pancreatic tumor in this study consisted of multiple transformed cell types, even though the sequence data were consistent with a predominantly epithelial origin (with some dedifferentiation; see below). The nonspecificity issue might be addressed in the future with a modified transgenic pig in which expression of the *KRAS*/*TP53* transgenes would be restricted to pancreatic ductal epithelium, or an epithelial-targeted AdCre vector (see below).

Exomic analysis of the OCM pancreatic tumors confirmed the presence of the *KRAS*^G12D^ and *TP53*^R167H^ transgenes, along with additional mutations in *KRAS*, *TP53* and *SMAD4* in some of the OCM subjects (2/4, 3/4 and 1/4 Oncopigs, respectively). In human PDAC, mutations in *KRAS*, *TP53* and *SMAD4* occur in 74%, 53% and 23% of patients, respectively (Table S5). The small number of OCM tumors (*n*=4), however, did not permit formal comparison of mutational rates between humans and Oncopigs.

Transcriptomic analysis of the OCM pancreatic tumors confirmed expression of the *KRAS*^G12D^ transgene and was consistent with expression of *TP53*^R167H^. Of note, the *TP53* probes were not targeted to the R167H point mutation, so both WT and mutant *TP53* gene transcripts would have been identified. We cannot rule out that the increase in total *TP53* transcript level was secondary to induction of the WT sequence (for example, in response to expression of the *KRAS*^G12D^ transgene). Transcriptomic analysis also demonstrated increased proliferative and EMT profiles, and upregulation of acidic mucins and IL-4/IL-13 signaling in these tumors. Human PDAC has a fibroinflammatory tumor microenvironment, with high levels of mucins, interleukins and EMT-associated TIMPs (Slapak et al., 2020; Tjomsland et al., 2016; Wang et al., 2017; Aiello et al., 2018), which all are associated with PC progression and metastasis (Shi et al., 2021; Hallett et al., 2012). OCM tumors also had reduced expression of pancreatic precursor and digestion/absorption genes, suggesting some tumor dedifferentiation.

It could be relevant to point out that transgene expression in the OCM is driven by the CAG promoter, whereas the analogous transgenes in the murine KPC model are under endogenous control, as noted in the Introduction. This difference in transcriptional control likely produced higher relative expression of the transgenes in the OCM with respect to the KPC model, which might help explain the aggressive and fulminant pancreatic tumor development that was observed in the OCM.

The presence of regional surface (‘drop’) metastases in the OCM subjects suggested that intraperitoneal spillage of AdCre during the injection procedure could have occurred, with resultant formation of peritoneal tumor nodules. This incidental finding may have implications in the development of an OCM-based model of peritoneal carcinomatosis. With respect to tissue-specific induction, we are planning future experiments (including the above-mentioned dose–response study) that will utilize an adenoviral vector in which Cre recombinase expression is driven by the keratin 8 promoter (Tao et al., 2014), which should limit mutant gene activation to the pancreatic epithelium. With regard to the cytokine arrays, OCM subjects with tumor did show elevation of a cytokine subset, but these relative increases were not significantly different from those in subjects without tumor. This study was limited by availability of OCM litters, which, for example, resulted in the unbalanced OCM male:female ratio (Table 1).

AdCre injection into the connecting lobe of the KRAS/p53 Oncopig generated pancreatic tumor with a frequency of 71%; OCM tumors were predominantly epithelial on histology, but less differentiated with respect to gene expression. However, OCM pancreatic tumors contained mutations and transcriptomes that resembled human PC. The fulminant and rapidly fatal course of these tumors may require refinement in the induction parameters so that a more tractable porcine PC can be obtained.

## MATERIALS AND METHODS

### Experimental subjects and design

Transgenic Oncopigs (LSL-*KRAS*^G12D^-IRES-*TP53*^R167H^) and their WT littermates were purchased from the National Swine Research and Resource Center (NSRRC) at the University of Missouri Columbia (nsrrc.missouri.edu). The OCM subjects were a hybrid of Minnesota minipigs and domestic pigs. The genotype of each porcine subject was confirmed with PCR upon subject delivery (Fig. S1). Swine were housed two littermates per pen, except for 1[Supplementary-material sup1]week of individual housing post-laparotomy (but with contact through the pen grating), in order to prevent wound cannibalism. Swine were fed *ad libitum* with standard hog feed (Purina Nature's Match® Sow and Pig Complete Feed; www.purinamills.com). The basic experimental design included a ≥1 week acclimatization period after subject delivery to the research facility. Each subject then underwent an induction procedure (laparotomy under general anesthesia; one major survival procedure), followed by observation for up to 3 months.

### Survival procedure: tumor induction

#### Laparotomy and exposure

A 15 cm upper midline laparotomy incision was made. Abdominal wall retraction was maintained with a self-retaining abdominal (Bookwalter) retractor. The long tongue of the spleen was placed into the right upper quadrant. The small intestine was held inferiorly with laparotomy pads and the self-retaining retractor. The pylorus was identified, grasped and brought up to the incision. The proximal pancreas could be elevated with the pylorus and proximal duodenum with minimal dissection; this maneuver provided access to both the anterior and posterior surface of the proximal pancreas (see Results and Discussion). The colon typically was lightly adherent to the anterior surface of the pancreas with loose connective tissue, so the colon was mobilized inferiorly from the pancreas with scissors.

#### Injection of tumor induction reagent

There were three basic techniques for injecting the tumor induction reagent into the pancreas: (1) injection into the main pancreatic duct with parenchymal injections; (2) injection into the duct of the connecting lobe of the pancreas; and (3) technique 2 plus parenchymal injections. The induction reagent consisted of AdCre (adenovirus expressing Cre recombinase through the CMV promoter, serotype 5) at a concentration of 1×10^10^ pfu/100 µl (in saline), with an injection volume of 200 µl. Some subjects (see Results and Discussion) also received porcine IL-8 (5 ng/ml in the same 200 µl volume, mixed in with the AdCre and given as one injection).

#### Technique 1: main duct+parenchyma

After the above exposure of the pancreas, a 3 cm longitudinal enterotomy was made on the anti-mesenteric side of the duodenum, directly opposite the termination of the duodenal lobe of the pancreas into the duodenal wall, which was the location of the papilla of the main pancreatic duct. The incised duodenal walls were retracted laterally with silk stay sutures. The main pancreatic duct was then directly cannulated with a 20-gauge plastic catheter (Angiocath™ IV Catheter; Becton Dickinson) and injected with the induction reagent. Parenchymal injections of the same induction cocktail were also performed in some subjects on the anterior and posterior side of the proximal duodenal lobe of the pancreas (typically two injections on each side, using a 25-gauge needle). Each parenchymal injection site was marked with small dot (1-2 mm wide) of India ink, using a 27-gauge needle. The duodenal enterotomy then was closed in two layers, using a running 3-0 polyglactin 910 suture for the inner row, full thickness, inverting (Connell) technique. This was followed with an outer seromuscular row of interrupted 3-0 silk sutures (Lembert technique).

#### Technique 2: connecting lobe.

After exposure of the pancreas was obtained, the connecting lobe of the pancreas was identified where it joined the proximal duodenal lobe (see Results and Discussion). The connecting lobe then was doubly clamped and transected ∼1 cm inferior to the junction with the duodenal lobe. The proximal stump of the connecting lobe (against the duodenal lobe) was tied off with a 3-0 silk ligature. Using operative loupe (3.5×) magnification, the open end of the primary duct running through the connecting lobe was found in the free cut end of that lobe (see Results and Discussion). This duct was cannulated with a 20-gauge plastic catheter, and the induction reagent was injected into the duct of the connecting lobe. The catheter was withdrawn, and the free end of the connecting lobe then was immediately ligated with 3-0 silk.

#### Laparotomy closure

The abdominal incision was closed anatomically in layers, with running 3-0 polyglactin 910 in the peritoneum and posterior sheath, running 0-polydioxanone in the anterior aponeurosis, 3-0 polyglactin 910 in the panniculus carnosus and 4-0 polyglactin 910 in the dermis. Cyanoacrylate glue was applied over the skin incision; no other incisional dressing was applied. The animal's recovery from anesthesia was monitored until the subject was awake and mobile. Subjects were given half feeds on post-induction day 1 and were placed back on *ad libitum* feeds by day 2.

### Additional methods and materials

See Supplementary Materials and Methods for conduct of animal experiments as guided by Animal Research: Reporting of In Vivo Experiments (ARRIVE) standards, description of animal welfare concerns, animal numbers and randomization, anesthesia and analgesia, euthanasia, histology, serum testing, sequencing and statistical analysis, and other methods. All antibodies used in this study are listed in Table S10. The sample sizes in the different experimental groups are provided in Table S11.

## Supplementary Material

10.1242/dmm.049699_sup1 Supplementary information
