# Supplementary material for: Induction of pancreatic neoplasia in the KRAS/TP53 Oncopig
Source: Dis Model Mech. 2023 Jan 16;16(1):dmm049699. doi: 10.1242/dmm.049699 (PMC9884120; doi:10.1242/dmm.049699)
Supplement: Supplementary information [file dmm-16-049699-s1.pdf]

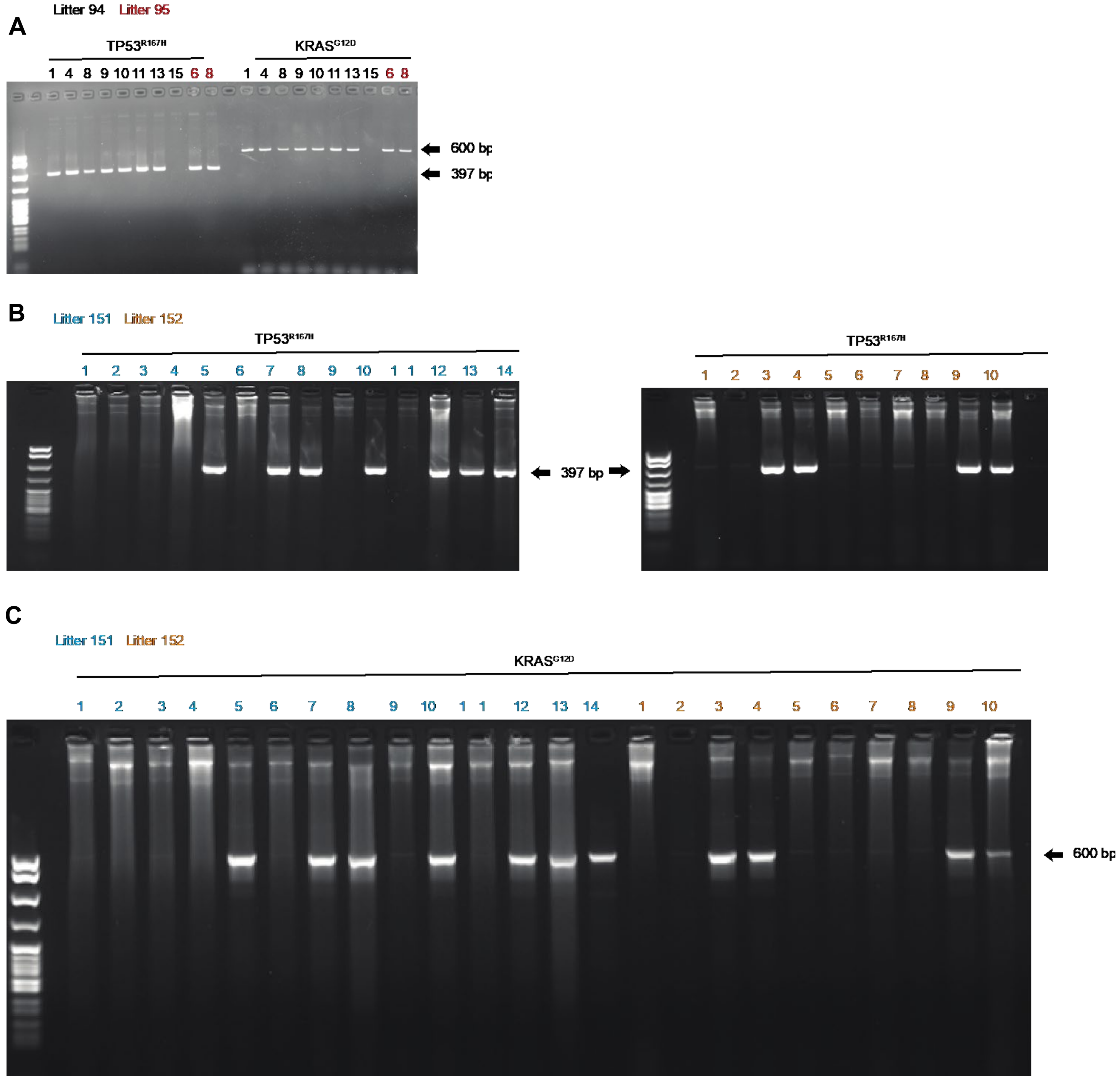

Fig. S1. Genotypes of WT and OCM subjects by PCR analysis. (A) Litter 94 and 95. (B, C) Litter 151 and 152.

Disease Models & Mechanisms • Supplementary information

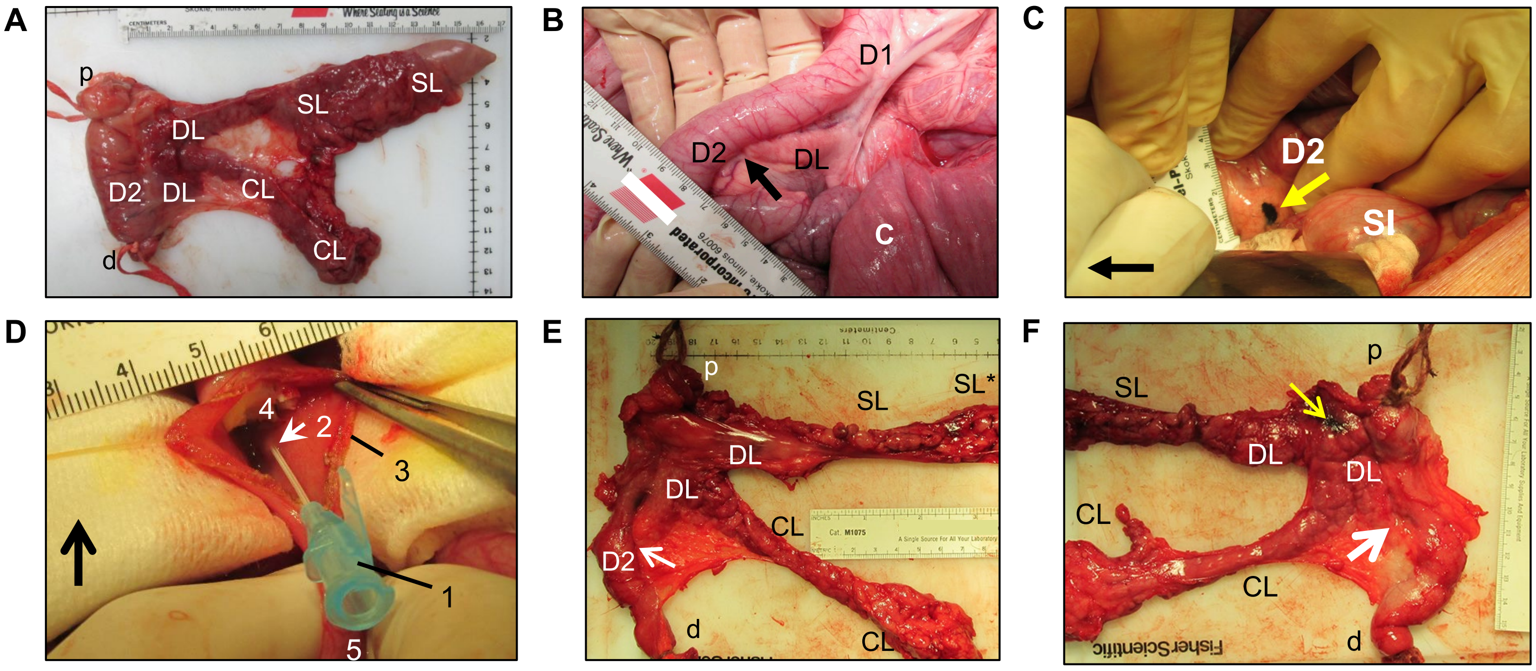

Fig. S2. Preliminary Oncopig Studies. A. Porcine pancreatic anatomy *ex vivo*. For reference, a view of the anterior surface of an explanted intact porcine pancreas (with duodenum) from a wild type domestic pig is shown. The porcine pancreas grossly consists of three lobes: duodenal, connecting, and splenic (*Transplantation* 2008;86: 1503). D2 = 2nd portion of duodenum; p = proximal duodenum; d = distal duodenum; DL = duodenal lobe of pancreas; SL = splenic lobe of pancreas; CL = connecting lobe of pancreas. Scale = cm. **B. Porcine pancreatic anatomy *in situ.*** View of porcine pancreas *in situ* in a live Oncopig, anterior surface, cephalad is at top of image. D1 = 1st portion of duodenum; D2 = 2nd portion of duodenum; DL = duodenal lobe of pancreas; C = colon; Arrow =

AdCre injection at two sites within the pancreas (4 x 10^8 viral particles in 100 µL per site). Under general anesthesia (refer to Methods in main manuscript), the pancreas was exposed via a ventral vertical midline incision. The 2nd portion of the duodenum (D2) has been elevated and retracted medially, exposing the posterior surface of the duodenal lobe of the pancreas. The yellow arrow indicates an India ink tattoo, which has marked Cre injection site no. 1, on the posterior surface of the duodenal lobe of the pancreas. SI = small intestine; scale = cm; black arrow = cephalad. **D. AdCre injection, site no. 2 (pancreatic duct).** Through the same midline laparotomy incision used in panel *c*, a 3 cm longitudinal duodenotomy was made on the antimesenteric side of the 2nd portion of the duodenum, opposite from where the main pancreatic duct entered the duodenum (refer to panel *b*). The same dose of AdCre (4 x 10^8 viral particles in 100 µL) was injected into the lumen of the main pancreatic duct (site no. 2), using a 22 g Angiocath (1) that was inserted through the duodenal papilla (2) of the pancreatic duct. 3 = Cut edge of the duodenotomy; 4 = lumen of proximal duodenum, showing small sponge temporarily placed to staunch the flow of gastrobiliary contents during the injection procedure; 5 = antimesenteric side of the distal duodenum; black arrow = cephalad. After injection, the duodenotomy was closed longitudinally in two layers with running 3-0 polyglactin 910 suture. Scale = cm. **E. Explanted Oncopig pancreas at necropsy, anterior surface.** After laparotomy with injection of AdCre, all five subjects recovered uneventfully and were tolerating regular feed within 3-4 days. There were no perioperative complications. Subjects were fed ad lib with no specialized husbandry for 4 mo. Average weight gain during the 4 mo observation period was 30.9 ± 8.4 kg (Table S2); all subjects were thriving and otherwise normal appearing. Serum laboratory testing just prior to euthanasia revealed small differences from reference values derived from 3-4 mo old domestic pigs (Table S3). Subjects underwent euthanasia with full necropsy (head, chest, abdomen) at 4 mo post-injection of AdCre (subject age 9-10 mo). Adhesions involving the operative site were present in all subjects, but there were no gross tumors, neither locally nor distantly. The explanted specimen in this panel (4 mo post-injection of AdCre) shows the anterior side of the pancreas. D2 = 2nd portion of duodenum; DL = duodenal lobe of the pancreas; CL = connecting lobe of the pancreas; SL = splenic lobe of the pancreas; SL* = region of splenic lobe used as negative control tissue for histology; p = proximal end of duodenal specimen; d = distal end of duodenal specimen; arrow = location of main pancreatic duct insertion into duodenum (AdCre injection site no. 2). **F. Explanted Oncopig pancreas at necropsy, posterior surface.** Other side of specimen shown in panel *e*. Small yellow arrow = India ink tattoo, indicating AdCre injection site no. 1. Large white arrow = location of main pancreatic duct insertion into duodenum (AdCre injection site no. 2). Scale = cm.

Disease Models & Mechanisms • Supplementary information

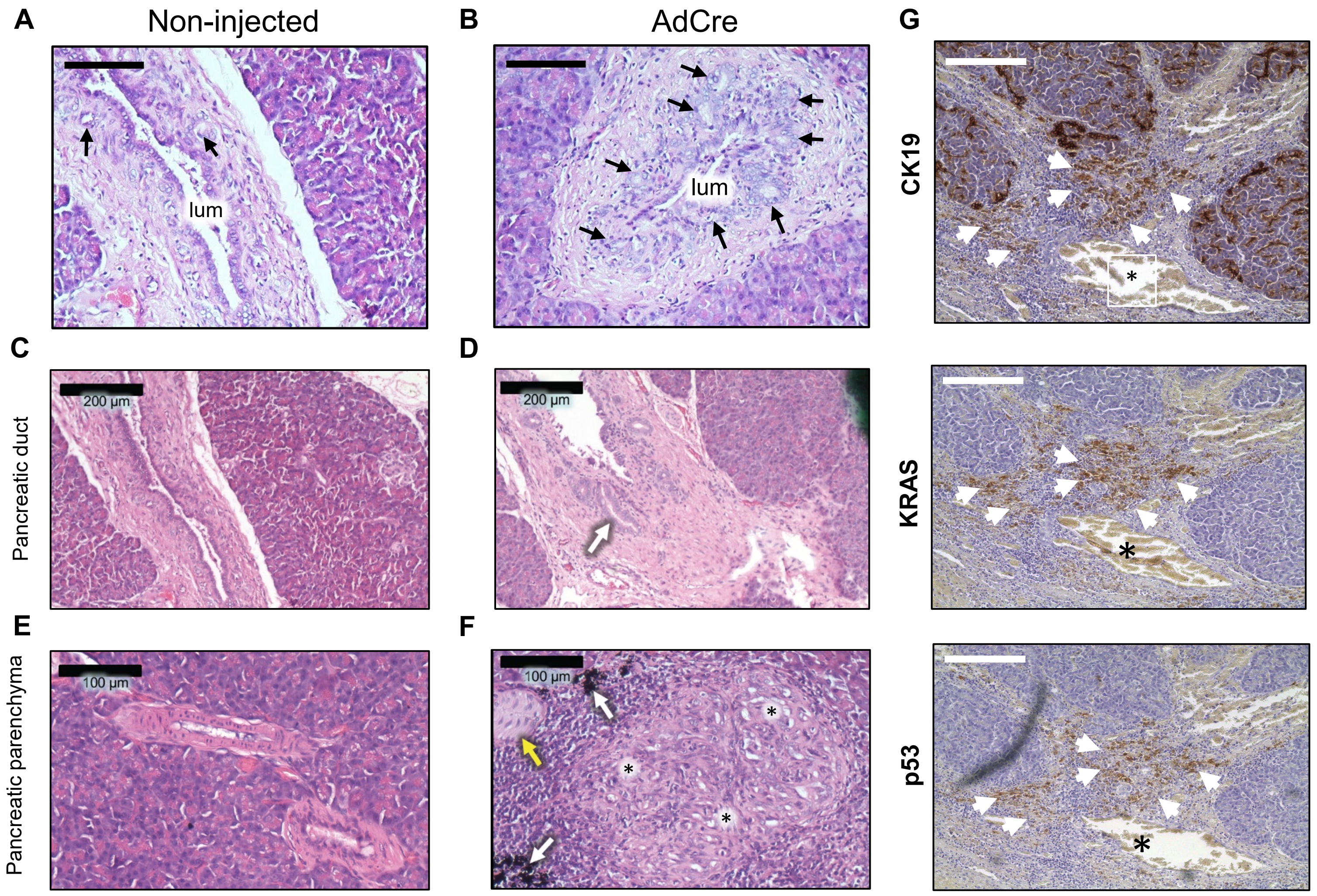

Fig. S3. Preliminary Oncopig Studies (continuation of Fig. S2).

(A & B) H&E images of pancreatic duct (AdCre site no. 2) from AdCre-injected Oncopigs. Explanted Oncopig pancreas from all five subjects was fixed in formalin and then underwent serial slicing at the two AdCre injection sites (site no. 1 = parenchymal, within the duodenal lobe; site no. 2 = into the lumen of the main pancreatic duct) to search for sub-centimeter tumors; none were found. Pancreas from the distal splenic lobe (see panel *A*) was used for negative control tissue (i.e., pancreas with no AdCre injection). That is, each Oncopig served as its own control. Representative images are shown. No lesions within the epithelium of the main pancreatic duct proper were observed. However, there appeared to be proliferation of accessory ducts around the main pancreatic duct from regions that had been exposed to AdCre (see arrows in panel *B*). In contrast, the pancreatic duct from the non-injected region (*A*) had relatively few accessory ducts (arrows) around the main duct. Bar = 1,000 µm; lum = lumen of main pancreatic duct. **(C-F)** Similar to panels *A-B*, the non-injected (negative control) tissue derived from the splenic lobe of the pancreas, a region of the organ physically distanced from the injection sites. In the non-injected regions (*C, E*), the morphology of the parenchyma and ducts was unremarkable. Around the AdCre-treated pancreatic duct, however, there appeared to be proliferation of the accessory ducts (arrow in panel *D*). In addition, there were proliferative-desmoplastic lesions at the parenchymal injection site, an example of which is shown in panel *I* (denoted with asterisks). White arrows in panel *D* = India ink marker; yellow arrow = pancreatic islet. **G. CK19, KRAS, and p53 Immunohistochemistry in AdCre-injected Oncopigs.** Paraffin blocks which had revealed proliferative-desmoplastic lesions within the Onco-pig pancreatic specimens were re-sectioned; consecutive sections then underwent immunohistochemistry as shown, with DAB as the primary stain and hematoxylin as the counterstain. In this series of three consecutive histologic sections, a proliferative-desmoplastic lesion is present in the middle of each image, surrounded by tongues of normal-appearing pancreatic parenchyma. A pancreatic duct (*) is present adjacent to this lesion. Regions within this lesion had overlapping positivity for all three antigens (CK19, KRAS, and p53), as indicated by the small black arrows. Bar = 200 µm.

Disease Models & Mechanisms • Supplementary information

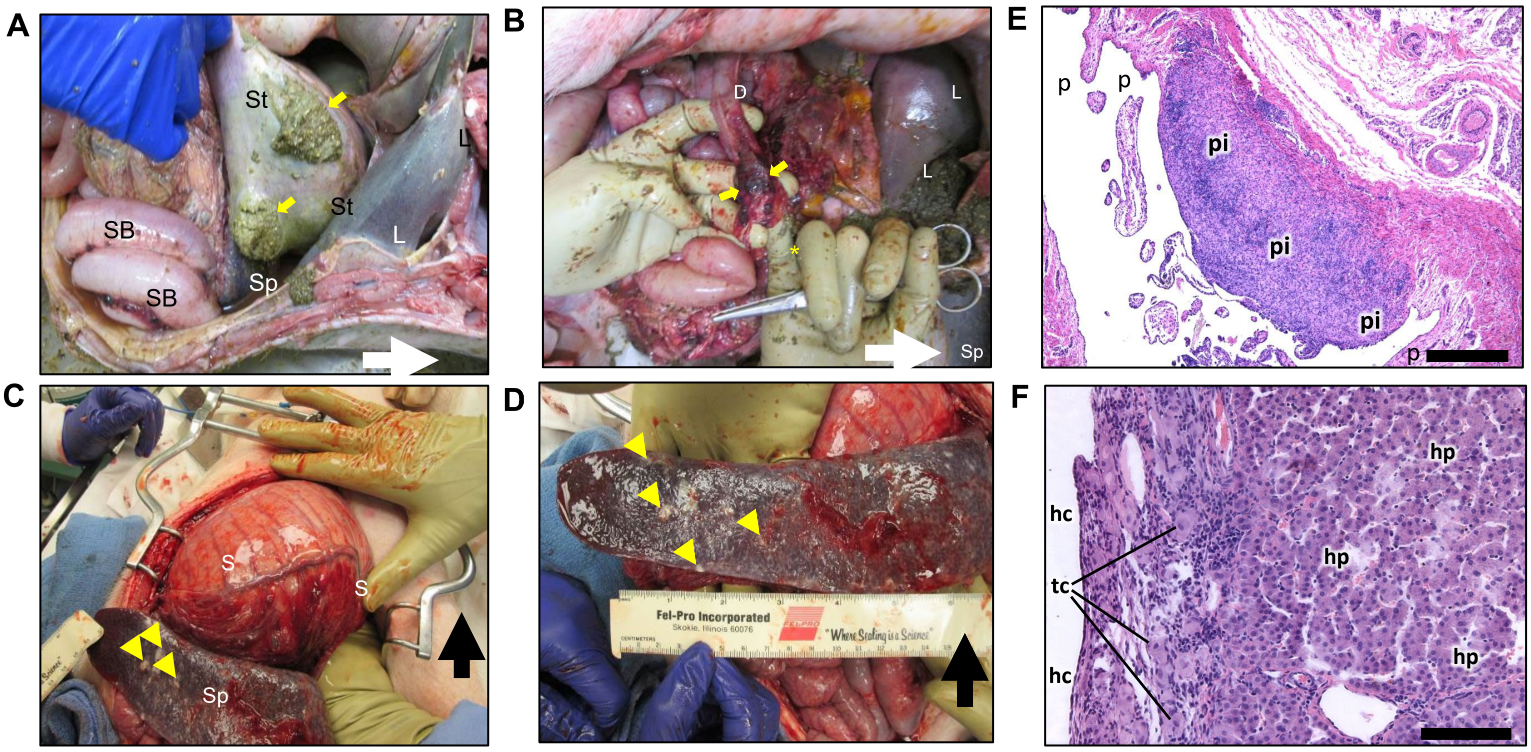

Fig. S4. Pancreatic tumor in Oncopigs (A) Pig expired in evening of postoperative day 18, after 1 wk of lethargy & poor po intake. Upon re-entering the abdomen at necropsy, ~1L of clear-brown ascites was encountered. Two adjacent perforations were found in the fundus of the stomach (yellow arrows), and copious amounts of gastric contents had spilled into the peritoneal cavity. The stomach was still distended with feed, however. There was gross evidence of peritonitis associated with this perforation, which suggested that it occurred while the subject was alive, making perforation the most likely immediate cause of death. The stomach and pylorus were opened with a longitudinal incision. The pylorus was patent and the segment of duodenum where the anastomosis was located was identified. The suture line of the anastomosis was intact, patent, and without inflammation or reaction. Distal to that was a hemorrhagic segment of bowel with a mass effect (B), adjacent to the site of location of the pancreatic injection. This section of bowel was not patent (i.e., it was the site of the obstruction which produced the gastric distention which led to the perforations. The rest of the pancreatic parenchyma felt firm and was hemorrhagic. The subject had lab testing 24 h prior to expiration: WBC = 17K; Hb = 13.7; plt = 255K; glucose = 134; bicarbonate = 40; creatinine = 2.0; amylase = 563; AST = 41; Tbili = 0.2. **(B)** Site of duodenal obstruction with mass effect. Finger (*) is shown inserted into proximal duodenum, but it is unable to traverse the distal duodenum at location of obstruction (yellow arrows). D = duodenum (distal, decompressed); large white arrow = cephalad. **(C)** This subject was euthanized 14 days after the tumor induction procedure because of inability to eat. At the induction procedure, the pancreas was exposed through an upper midline incision. An injection into the duct of the connecting lobe then was performed. The stomach (S) was distended with feed. The spleen (Sp) had multiple whitish plaques (yellow arrows). Similar to previous subjects, the primary cause of death in this Oncopig appeared to be hemorrhagic pancreatitis which produced extensive intraabdominal inflammation, with functional gastric outlet obstruction (ileus) and failure to thrive. (D) Necropsy. Close-up view of the spleen, demonstrating multiple flat, whitish plaques (yellow arrows), 1-5 mm in diameter. Large black arrow = cephalad. (E) Peritoneal implant from anterior abdominal wall > 10 cm away from pancreatic connecting lobe (i.e., from the AdCre injection site). Bar = 100 µm. (F) Hepatic capsule implant > 10 cm away from pancreatic connecting lobe. Bar = 500 µm.

Key: L = liver; SB = small bowel; Sp = spleen; St = stomach; small yellow arrows = perforations in the gastric fundus; large white arrow = cephalad. hc = hepatic capsule; hp = hepatic parenchyma; p = peritoneum; pi = peritoneal implant on abdominal wall; tc = tumor cell.

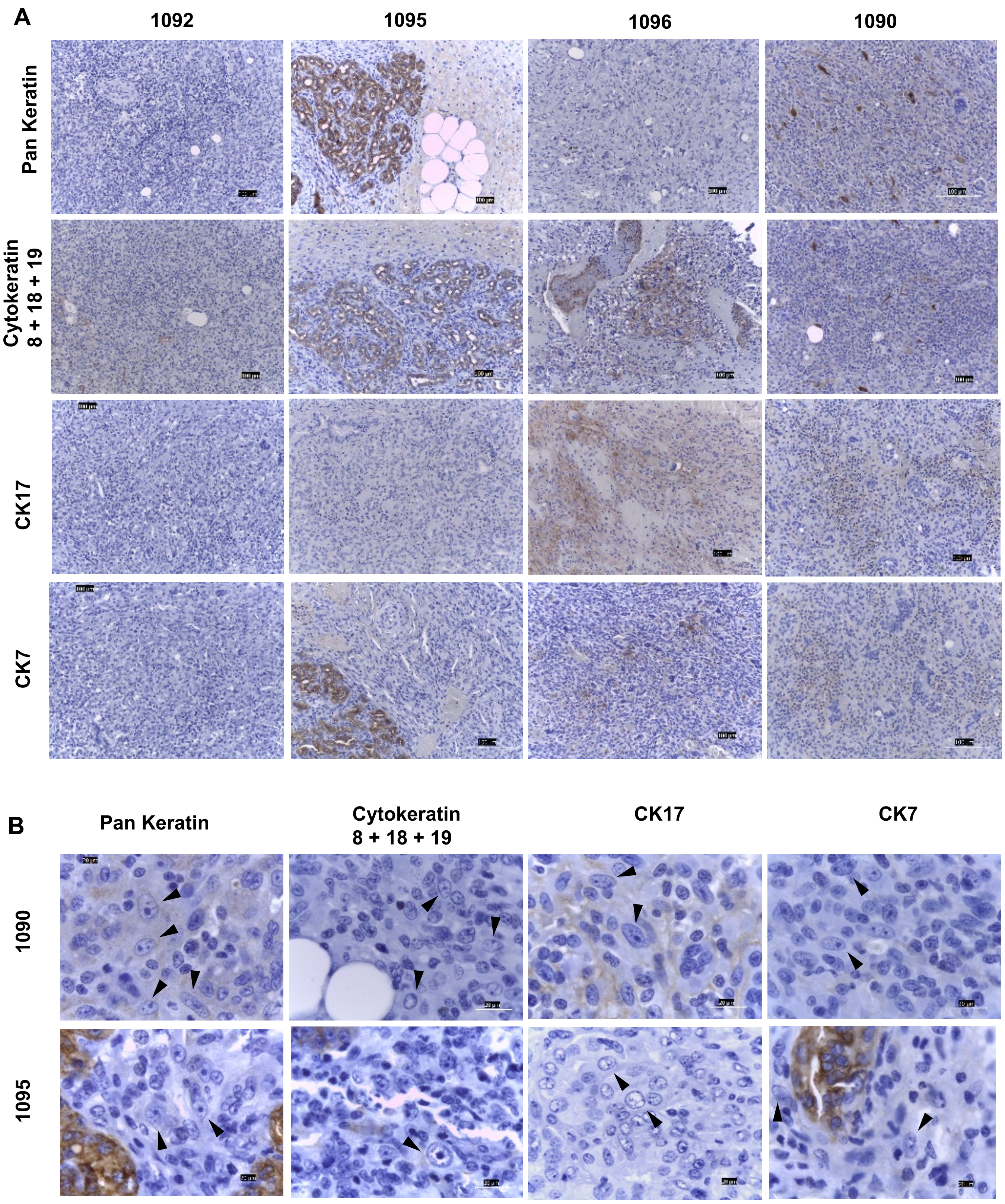

Fig. S5. Immunohistochemistry of tumors. (A) IHC of different cytokeratin markers did not identify tumor cells in the OCM pancreatic tumors. **(B)** Representative enlarged pictures shows that the tumor cells (arrowhead) are not expressing the protein markers.

Disease Models & Mechanisms • Supplementary information

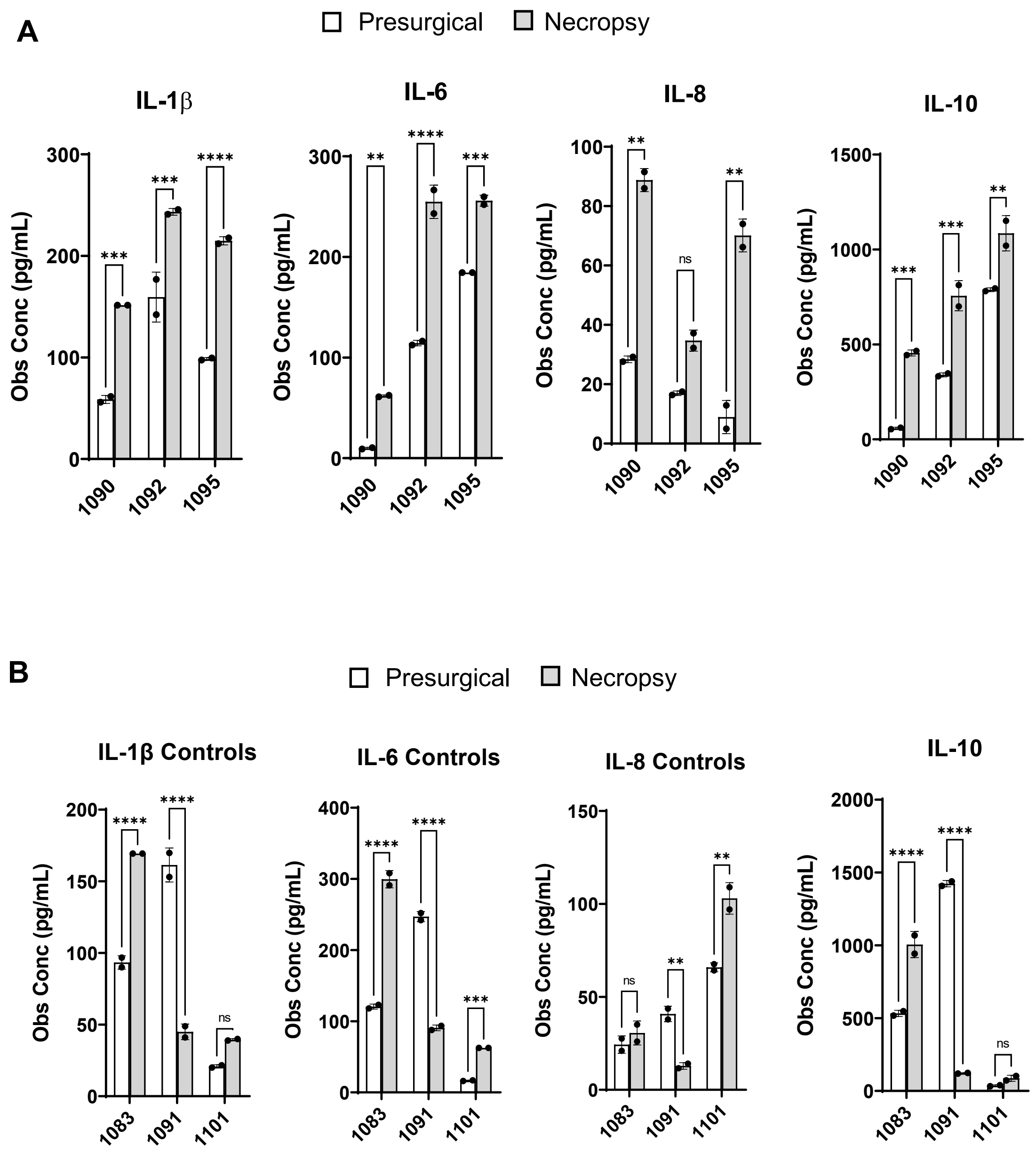

Fig. S6. Serum cytokine analysis (IL-1β, IL-6, IL-8, IL-10) comparing pre-induction (pre-surgery) *vs.* necropsy levels for OCM and WT subjects undergoing the tumor induction procedure. (A) OCM subjects (no. 1090, 1092, and 1095). **(B)** WT control subjects (no. 1083, 1091, and 1101). OCM pigs 1090 and 1095 received both AdCre and IL8 injections, while 1092 did only receive AdCre but not IL8 injection. In the control pigs, 1083 and 1091 are wild type pigs who had received both AdCre and IL8 injection, while 1101 was a OCM pig which only received IL8 but not AdCre injection. Each bar represents the mean ± SD of a duplicate array; Two-way ANOVA, ns = not significant; **p <0.01, ***p <0.001, ****p <0.0001.

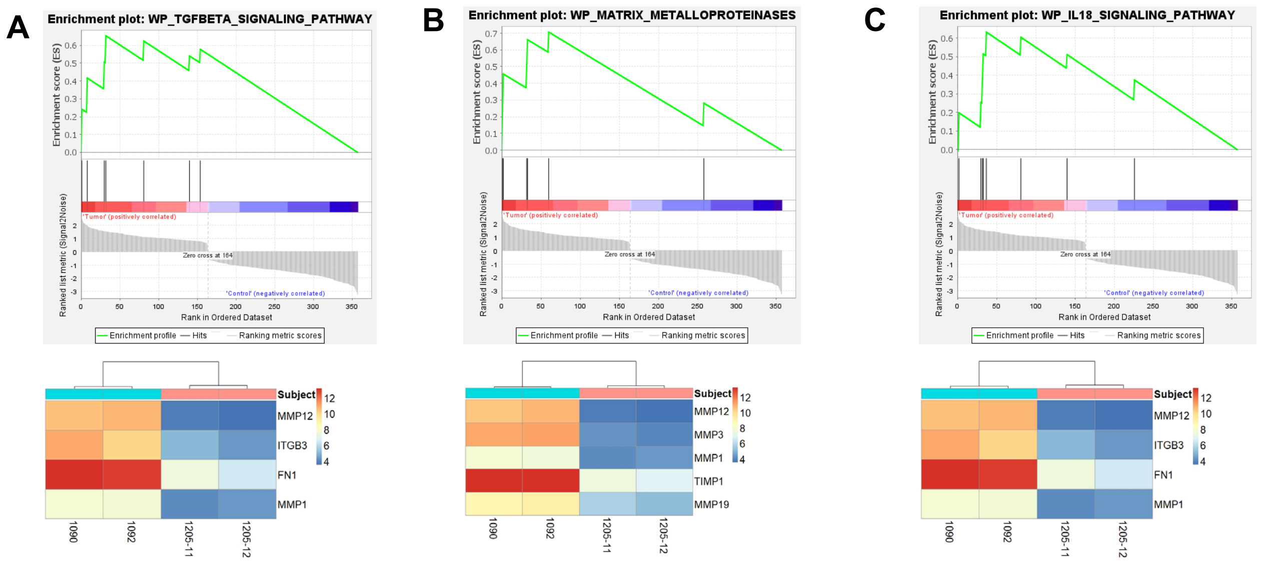

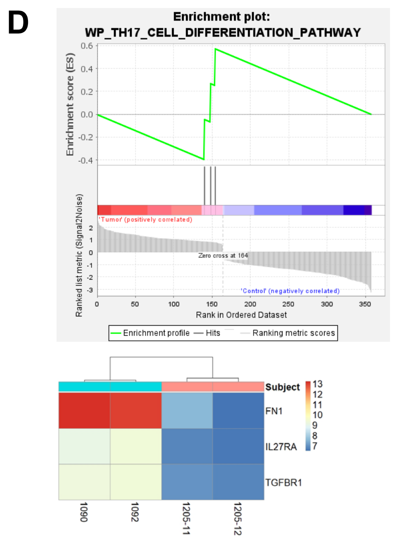

E

Gene sets	ES	NES
WP_TGFBETA_SIGNALING_PATHWAY	0.65397	2.39475
WP_MATRIX_METALLOPROTEINASES	0.70697	2.03708
WP_IL18_SIGNALING_PATHWAY	0.63227	1.84781
WP_TH17_CELL_DIFFERENTIATION_PATHWAY	0.57183	2.0949

Fig. S7. Gene set enrichment analysis of the tumor microenvironment. Enrichment plots of the tumor microenvironment related gene sets (A) TGF-beta, (B) matrix metalloproteinases, (C) IL18 signaling and (D) TH17 signaling pathways. Expression heatmap of the enriched genes were shown below each enrichment plots. (E) Enrichment score (ES) and Normalized Enrichment Score (NES) of the shown enriched gene sets. All enriched gene sets were statistically significant after false discovery rate correction of the p-values (<0.001).

Disease Models & Mechanisms • Supplementary information

Table S1. Porcine descriptive data

Click here to download Table S1

Table S2. Descriptive data of preliminary Oncopig studies

Click here to download Table S2

Table S3. Preliminary Oncopig Studies: Serum laboratory testing just prior to euthanasia for Oncopigs

Click here to download Table S3

Table S4. Cytokine data

Click here to download Table S4

Table S5. All high-impact variations in OCM tumors

Click here to download Table S5

Table S6. New and existing variations in major pancreatic cancer genes of OCM tumors

Genes	Variations	Location	Type	Samples
KRAS	Deletion (GAT) with rs705858787	5:48548834-48548836	3 prime UTR variant, cDNA (4665-4667)	1095, 1096 vs OCM control and Control
	Deletion of rs701486753 (A)	5:48549683	Downstream gene variant	1095, 1096 vs OCM control and Control
	rs329769372 (G/A)	5:48504818	Upstream gene variant	1095 vs OCM control and Control
	Mutation (G/A)	5:48513419	Missense variant (G to D)	1090, 1092, 1095 & 1096 vs Control
TP53	rs336094858 (T/C)	12:52940975	Intron variant (T to C)	1092, 1095 and 1096 vs OCM control
	rs343411452 (T/C)	12:52942629	Intron variant (T to C)	1092, 1095 and 1096 vs OCM control
	rs81211696 (T/G)	12:52941073	Synonymous variant (T to G)	1090, 1092, 1095 & 1096 vs Control
	rs318253531(C/A)	12:52942206	Synonymous variant (C to A)	1090, 1092, 1095 & 1096 vs Control
	rs81211695 (C/T)	12:52942221	Synonymous variant (C to T)	1090, 1092, 1095 & 1096 vs Control
	rs334394956 (G/A)	12:52942644	Synonymous variant (G to A)	1090, 1092, 1095 & 1096 vs Control
	rs324980623 (G/A)	12:52942707	Synonymous variant (G to A)	1090, 1092, 1095 & 1096 vs Control
	rs345021946 (C/A)	12:52942996	Synonymous variant (C to A)	1090, 1092, 1095 & 1096 vs Control
	Mutation (T/C)	12:52943005	Intron variant (T to C)	1090, 1092, 1095 & 1096 vs Control
	Mutation (C/T)	12:52943212	Intron variant (C to T)	1090, 1092, 1095 & 1096 vs Control
SMAD4	Mutation (A/T)	1:100590467	Intron variant (A to T)	1090 vs OCM control and Control

Table S7. Alteration frequency of KRAS, TP53 and SMAD4 genes in human pancreatic cancer

Click here to download Table S7

Table S8. Variations and genetic aberrations in pancreatic cancer related genes in OCM tumors

**Genes**	**Number of variations**	**Type of variations**	**Samples**
AKT1	15	SNPs (15), rsID (0)	All samples
AKT2	12	SNPs (8), rsID (2) | single base splice insertion (2)	All samples (SNPs)
			All vs OCM Control (insertion)
AKT3	30	SNPs (24), rsID (24) | insertions (5), 3 UTR (2), intronic (3) | six base deletion, intronic, with SNPs (1)	All samples (SNPs)
			Insertions in 1090, 92 & 96 vs Control and OCM controls
ARAF	3	SNPs (3), rsID (3)	All vs controls, 1095, 1096 vs OCM controls
ARHGEF6	63	SNPs (59), rsID (59) | one 17bp insertions, intronic (1) | 3 deletions, downstream (2), intron (1)	All samples (SNPs)
			Insertion (1096 vs control, OCM control)
			Deletion (all samples)
BAD	18	SNPs (18), rsID (17)	All samples
BCL2L1	3	SNPs (3), rsID (3)	1095 vs control, all vs OCM Control
BRAF	34	SNPs (34), rsID (34)	All samples
CASP9	106	SNPs (100), rsID (1)	All samples
		13 bp intronic insertions (1)	Insertions in 1096 vs Control and OCM control
		Deletions (4): intronic (3), 3 UTR (1)	Deletions in 1095, 1096 and 1090
CCND1	5	SNP (4), rsID (2) | Insertion single base intronic (1)	All samples
			Deletion: 1090, 92, 96
CDK4	23	SNP (23), rsID (21) | 9 bp insertion intronic (1)	All samples
			1095, 1096 vs Control, OCM control
CDK6	17	SNP (17), rsID (11) | single bp deletion (2), double bp deletion (1), 4 bp deletion (1)	All samples
			1090, 1095 vs Control and OCM control
ERBB2	15	SNPs (13), rsID (13), 5 downstream, 6 intronic, 1 missense, 2 synonymous, 1 upstream | insertion (2)	All samples
RAD51	26	SNPs (26), rsID (25), 11 3 UTR, 10 introns, 1 missense, 1 synonymous, 3 downstream	All samples

SNPs: Single nucleotide polymorphisms, rsID: Reference SNP ID, UTR: Untranslated region

Disease Models & Mechanisms • Supplementary information

Table S9. List of over-expressed and under-expressed genes in OCM tumor transcriptomics

Click here to download Table S9

Table S10. Information on antibodies used for immunoblotting and immunohistochemistry

Antigen	Product No.	Company	Website	Type	Host	Clone number
Cytokeratin 19	ab7754	Abcam	Abcam.com	monoclonal	mouse	A53-B/A2
Mutant KRAS^{G12D}	GTX132407	Genetex	Genetex.com	polyclonal	rabbit	
Mutant p53	Bsm-54279R	Bioss	Biossusa.com	monoclonal	rabbit	2F6
Ki67	ab16667	Abcam	Abcam.com	monoclonal	rabbit	SP6
Vimentin	677801	Biolegend	Biolegend.com	monoclonal	mouse	O91D3
CD31	MCA1746GA	Bio-Rad	Bio-rad-antibodies.com	monoclonal	mouse	LCI-4
Pan-Keratin	4545S	Cell Sig.	Cellsignal.com	monoclonal	mouse	C11
Cytokeratin 8+18+19	ab41825	Abcam	Abcam.com	monoclonal	mouse	2A4
Cytokeratin 7	ab9021	Abcam	Abcam.com	monoclonal	mouse	RCK105
Cytokeratin 17	bsb2729	BioSB	Biosb.com	monoclonal	mouse	BSB-33

Table S11. Sample size in different experimental groups

Type of experiment		Number of Oncopigs	Number of wild type pigs
Control, no Ad-Cre injection		2	2
Test, Ad-Cre Injection	Main pancreatic duct (Technique 1 or MPD/T1)	2	0
	Pancreatic connecting lobe injection (Technique 2 or CL/T2)	12	4
Transcriptome of the normal porcine pancreas		0	2

Supplementary Materials and Methods

Detailed procedure protocols are available from the senior author (MAC) upon request.

Survival Procedure: Set-Up and Anesthesia

Swine were fasted for 24 h prior to the procedure, with free access to water. On day zero, subjects were weighed, and underwent induction with ketamine (2.2 mg/kg), Telazol® (1:1 w/w tiletamine:zolazepam, 4.4 mg/kg) and xylazine (2.2 mg/kg), given as a single IM (intra-muscular) injection. Buprenorphine SR (0.2 mg/kg) was given as an IM injection into the right hip. EKG, pulse oximetry, and lingual end-tidal CO_2 monitors were placed and connected to a BM5 Bionet monitor (bionetus.com). Endotracheal intubation was performed with a 6-7 mm ID (internal diameter) tube. The subject rested on a water-circulated warming blanket that was set at 102˚F. An auricular IV (intravenous) line was placed, and LR (Lactated Ringers solution) was administered at 500 mL/h. Cefovecin sodium (Convenia®; 8 mg/kg IM) and Buprenorphine SR (0.2mg/kg SC or subcutaneous) were administered during the induction period. Anesthesia was maintained with isoflurane (1-2%) and supplemental oxygen (3-5 L/min) using a Matrx® ventilator (midmark.com). The ventilator rate initially was set at 12-15 breaths per minute with a tidal volume of 8 mL/kg, and subsequently adjusted to maintain the EtCO2 at 40-50 mm Hg. Phlebotomy was performed on a forelimb or auricular vein. The ventral abdomen, groins, and thorax were scrubbed with chlorhexidine soap, and then depilated with electric clippers. Cotton blankets were placed over non-surgical areas to minimize subject heat loss. The final abdominal preparation was performed with alcohol-based chlorhexidine (ChloraPrep™; bd.com), and then the upper midline region was sterilely draped.

Reagents

Ad5CMVCre-eGFP (AdCre) was purchased from the University of Iowa Vector Core (vector-core.medicine.uiowa.edu). Porcine IL-8 was purchased from Novus Biological (NBP2-35234; novusbio.com). General chemicals were purchased from Millipore Sigma (www.sigmaaldrich.com). All the antibodies that were used in this study are listed in Table S10.

Standards, Rigor, Reproducibility, Transparency, Power, and Randomization

To the extent possible, the animal studies of this report were designed, performed, and reported in accordance with both the ARRIVE recommendations (Animal Research: Reporting of *In Vivo* Experiments; see Supplementary files)[1] and the National Institutes of Health Principles and Guidelines for Reporting Preclinical Research.[2] Due to the high cost and limited availability of the transgenic OCM subjects in this report, formal randomized dose-response experiments were not practical. For example, to determine whether there was a difference in tumor induction rate (yes/no state) between two different induction methods at $\alpha = 0.05$ and power = 0.8, then OCM groups size would be >30, which would be impractical from a cost and acquisition standpoint.

Animal Welfare Statement

The animals utilized for this report were maintained and treated in accordance with the *Guide for the Care and Use of Laboratory Animals* (8th ed.)[3] and in accordance with the Animal Welfare Act of the United States (U.S. Code 7, Sections 2131 – 2159). The animal protocol pertaining to this manuscript was approved by the Institutional Animal Care and Use Committee (IACUC) of the VA Nebraska-Western Iowa Health Care System (ID number 1124) and by the IACUC of the University of Nebraska Medical Center (ID number 19-053-FC). All procedures were performed in animal facilities approved by the Association for Assessment and

Disease Models & Mechanisms • Supplementary information

Accreditation of Laboratory Animal Care International (AAALAC; www.aaalac.org) and by the Office of Laboratory Animal Welfare of the Public Health Service (grants.nih.gov/grants/olaw/olaw.htm). All surgical procedures were performed under isoflurane anesthesia, and all efforts were made to minimize suffering. Euthanasia was performed in accordance with the AVMA Guidelines.[4] A table summarizing the sample size in different experimental groups are presented in Table S11.

Study Termination and Euthanasia

The prescribed post-induction observation period was three months. Criteria for early removal from the study and euthanasia were symptoms of failure to thrive (anorexia, lethargy, decreased movement, abnormal breathing, or other signs of distress) or sepsis (fever, wound disruption or drainage). At the time of euthanasia, each subject received sedation with an IM shot of ketamine/Telazol/xylazine, as described above, and then were endotracheally intubated. Inhalational isoflurane (5%) was administered via the ventilator. The prior midline incision was reopened. Inferiorly this incision was extended in paramedian fashion to avoid midline structures (such as the urethra in males). The completed necropsy incision extended from xiphoid process to the pelvic inlet. The bilateral thoracic cavity was entered by transversely incising the diaphragm just inferior to the xiphoid process. The intrathoracic portion of the inferior vena cava was easily identified as it emerged from the liver in the posterior mediastinum. Phlebotomy was performed from the cava, and then Fatal-Plus® (pentobarbital sodium, 390 mg/mL; 1 mL per 4.5 kg body weight) was administered by caval injection. Two minutes after administration of Fatal-Plus®, the inferior vena cava was transected just above the diaphragm to exsanguinate the subject. A gross necropsy involving the lungs, heart, liver, kidneys, pancreas, intestines, bladder, and associated peritoneal surfaces then was performed, with biopsy of any suspicious lesions.

Disease Models & Mechanisms • Supplementary information

Tissue processing, Histology and Immunohistochemistry

Tissue was either formalin fixed and sent for further processing, or fresh tissue was taken and digested for *in vitro* analysis (see below). Tissue that was cut from formalin-fixed samples was stained with H&E for pathological examination. IHC was performed by deparaffinizing slides and heating using citric acid antigen retrieval buffer (Vector laboratories, H3300). Following antigen retrieval, endogenous peroxidases were quenched with 3% hydrogen peroxide solution for 5 min. Blocking was done using 2.5% goat serum blocking buffer. Primary antibodies were incubated overnight at 4ºC and are listed in Table S6. Vector Laboratories (vectorlabs.com) ImmPress® goat anti-mouse (MP-7452) or anti-rabbit (MP-7451) IgG polymer kits were used for primary antibody detection per the manufacturer's instructions. Detection of the HRP/ peroxidase enzyme was performed with SignalStain® DAB Substrate Kit from Cell Signaling (cat. no. 8059; www.cellsignal.com), per the manufacturer's instructions. Alcian blue staining was performed using the Alcian Blue stain kit (pH 2.5, Mucin Stain) from abcam (cat. no. ab150662; www.abcam.com). For each slide, four different images were captured at 20X or 40X magnification and quantification was performed using open source software for digital image analysis (ImageJ; imagej.nih.gov/ij).

Cytokine Analysis

Blood was extracted with a syringe and needle before surgery and after necrops, and then immediately transferred to 10 mL heparinized tubes. Plasma was extracted per the Eve Technologies sample preparation protocol (evetechnologies.com; see Supplemental Information). Samples were sent to Eve Technologies for performance of a 13-plex (PD13) cytokine/chemokine array, which evaluated 13 different markers (IL-1α, IL-1β, IL-1ra, IL-2, IL-4, IL-6, IL-8, IL-10, IL-12, IL-18, GM-CSF, IFNγ, TNFα) using the Millipore MILLIPLEX

Disease Models & Mechanisms • Supplementary information

MAP Porcine Cytokine/Chemokine Magnetic Bead Panel (cat. no. PCYTMG-23K-13PX, www.merckmillipore.com). Samples were detected through a multiplexed immunoassay that was analyzed with a BioPlex 200 (Bio-Rad, www.bio-rad.com).

Exome sequencing and analysis

Exome sequences were captured from tumor genomic DNA (isolated by AllPrep DNA/RNA Mini Kit, Cat. No. 80204, www.qiagen.com) by SeqCap EZ HyperCap Exome capture probes, designed for pigs (Roslin Pig Exome Design Version 1) according to manufacture protocol (Roche Sequencing and Life Science, Wilmington, MA, USA), and sequenced on a NextSeq500 instrument (Illumina Inc., San Diego, CA, USA). The sequences were merged and trimmed using the fqtirm tool (https://ccb.jhu.edu/software/fqtrim) to remove adapters, terminal unknown bases (Ns), and low quality 3’ regions (Phred score < 30). Sequences then were processed by bcbio-nextgen 1.2.4 (https://doi.org/10.5281/zenodo.3564938) tool kit with QC (quality control) check by MultiQC 1.9, alignment by bwa, and variant calling by FreeBayes, GATK-HaplotypeCaller, and SAMtools. Variants were called ≥2 among the three variant callers to generate the final VCF (variant call format) file. The bcftool in SAMtools was used to generate a single common VCF file for each comparison group and was also used to generate a unique VCF file for each sample in each comparison. The VCF files were then subjected to VEP (variant effect predictor) tool to annotate and predict the effects of each variant. Finally, Circos was used to visualize the variants on pig reference genome. The exome sequencing data is available at SRA with accession number: PRJNA838612.

Transcriptomic analysis

Total RNA was isolated from normal pig pancreas and pancreatic tumor samples using AllPrep DNA/RNA Kits (Qiagen, Germantown, MD, USA), analyzed for RNA integrity score

Disease Models & Mechanisms • Supplementary information

(RIN) by TapeStation System (Agilent, Santa Clara, CA, USA), and then hybridized on a Porcine Gene 1.1 ST array (Thermo Fisher Scientific, Waltham, MA, USA). The data was quality checked, normalized, and analyzed on a Transcriptome Analysis Console (Agilent, Santa Clara, CA, USA). GESA (Gene Set Enrichment Analysis) was used to identify enriched expression signatures in the tumor samples. The gene expression microarray data is available at GEO with accession number: GSE203011.

Eve Technologies serum sample preparation protocol

1. Allow blood to clot for 30min or more at room temp. After clotting, centrifuge at 1000 x g for 10min at 4°C.
2. Aliquot serum immediately into a pyrogen/endotoxin-free polypropylene tube.
3. Store samples at ≤-20°C (for ~1 month storage life) or ≤-70°C (for more than 1 month storage).
4. Avoid using hemolyzed or lipemic sera.
5. Avoid multiple freeze/thaw cycles (>2 cycles).

Statistical Analysis

Data are reported as mean ± standard deviation. Comparison of means was performed using t-testing or two-way ANOVA as indicated, with the level of significance set at $p < 0.05$.

References (Fig. S2 only)

1. Kilkenny C, Browne WJ, Cuthill IC, et al. Improving bioscience research reporting: the ARRIVE guidelines for reporting animal research. PLoS Biol 2010;8:e1000412.
2. National Institutes of Health. Principles and Guidelines for Reporting Preclinical Research.
3. Committee for the Update of the Guide for the Care and Use of Laboratory Animals. Guide for the Care and Use of Laboratory Animals. . Washington, DC: The National Academies Press, 2011.
4. American Veterinary Medical Association Panel on Euthanasia. AVMA Guidelines for the Euthanasia of Animals: 2020 Edition. Schaumberg, IL: American Veterinary Medical Association, 2020.
